# Periodontitis and Platelets Status: A Systematic Review With Meta‐Analysis and Trial Sequential Analysis

**DOI:** 10.1111/jre.13398

**Published:** 2025-03-11

**Authors:** Dimitris Sokos, Marja L. Laine, Elena A. Nicu, Kelly Hiu Lam Chung, Ni‐ni Dong Qing Sluijk, Dagmar Else Slot, Sergio Bizzarro

**Affiliations:** ^1^ Department of Periodontology Academic Center for Dentistry Amsterdam (ACTA) University of Amsterdam and Vrije Universiteit Amsterdam Amsterdam the Netherlands; ^2^ Parofix Sibiu Romania

**Keywords:** periodontal diseases, platelet activation, platelet count, platelet volume

## Abstract

**Aim:**

Circulating platelets are essential in hemostasis, thrombosis, and immune responses. Modifications in platelet function may impact immunological reactions to dental biofilm and cardiovascular health. Understanding changes in platelet status and activity in patients with periodontitis is still a subject of investigation. This study aimed to synthesize evidence from observational studies that investigated platelet status and activity in patients with and without periodontitis.

**Methods:**

MEDLINE‐PubMed, EMBASE, and Cochrane‐CENTRAL Library databases were searched up to November 2024. Primary outcomes included platelet count (PC) and mean platelet volume (MPV). Secondary outcomes encompassed any other biomarker relevant to platelet status and activity. Methodological quality was evaluated using the Newcastle‐Ottawa scale, and heterogeneity was analyzed. Descriptive analysis of outcomes and meta‐analysis, incorporating trial sequential analysis of PC and MPV, were conducted. The body of evidence was graded by utilizing the Grading of Recommendations Assessment, Development, and Evaluation (GRADE).

**Results:**

3621 unique records were identified, resulting in 23 eligible studies with 22 variables evaluating the platelet status. Fourteen studies exhibited a low risk of bias, and 9 exhibited moderate risk of bias. PC in patients with periodontitis was significantly higher compared to individuals without periodontitis (Mean Difference (MD) = 23.55 ×10^9^/L, 95% CI [7.68; 39.43]). There was no statistically significant difference between the groups for MPV (MD = 0.16 fL, 95% CI [−0.49; 0.82]). Trial sequential analysis indicated a conclusive meta‐analysis of PC and highlighted the need for additional data on MPV from future trials.

**Conclusions:**

The certainty is moderate for slightly higher PC in patients with periodontitis compared to individuals without it and low for no difference in MPV between the two groups. The evidence is not robust to claim a clear difference in other platelet activation biomarkers between the two groups.

**Trial Registration:** International Prospective Register of Systematic Reviews (PROSPERO) by number: CRD42023439051

## Graphical abstract

The certainty is moderate for a slightly higher platelet count in patients with periodontitis compared to individuals without periodontitis and low for no difference in mean platelet volume between the two groups. The evidence is not robust to claim a clear difference in other platelet activation biomarkers between the two groups.

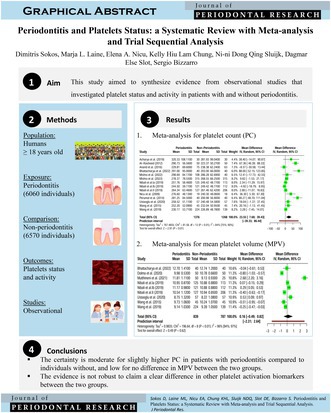

## 1 Introduction

Platelets are disk‐shaped, nonnucleated blood cells, exhibiting versatile functionalities, including involvement in hemostasis, thrombosis, and the immune system [1, 2, 3]. They facilitate the crosstalk between innate and adaptive immunity by activating antigen‐presenting cells and eliminating opsonized bacteria [3, 4, 5, 6]. Additionally, platelets serve as the first line of defense to locally control infection, contributing to immunothrombosis [7].

Platelet activation is implicated in pathological conditions like atherosclerosis, atherothrombosis, and subsequent coronary vascular and cerebrovascular diseases [8]. Various stimuli, including endothelium injury, hemodynamic alterations, circulating bacteria or their virulence factors, and inflammatory markers, can trigger platelet activation [3, 9, 10, 11].

Periodontitis is a chronic, complex inflammatory disease caused by a dysfunctional immune response triggered by the dental biofilm and marked by the degradation of the periodontal hard and soft tissues. Its main clinical manifestations include alveolar bone loss, gingival bleeding, and increased probing depth [12, 13].

Epidemiological evidence has linked periodontitis to an increased risk for cardiovascular diseases (CVDs) [14]. Oral bacterial species and their virulence factors enter the bloodstream through the ulcerated pocket epithelium, resulting in transient bacteremia. This takes place especially during periodontal therapy, but it may also occur during daily common oral hygiene or chewing activities [15, 16]. Additionally, periodontitis is associated with elevated levels of inflammatory biomarkers in the main bloodstream, resulting in a chronic low‐grade inflammatory state among affected individuals [17, 18, 19].

Considering the important role of platelets in the etiopathogenesis of CVDs, to biologically support the association with periodontitis, several studies have focused on the alteration of platelet status in patients with periodontitis [4, 20, 21]. Furthermore, the role of platelets in the immune response against the dental biofilm has been investigated. Previous ex vivo studies have shown an elevated platelet count (PC), along with the formation of platelet aggregates and platelet –leukocyte aggregates in the gingival crevicular fluid and inflamed gingival tissues of patients with periodontitis [22, 23, 24]. The elevated recruitment of platelets is attributed to the activation of Toll‐like receptors on their surfaces induced by components of the dental biofilm [25].

Therefore, the primary aim of this study was to synthesize the evidence from observational studies regarding the differences in platelet status and activity among patients with periodontitis compared to individuals without it.

## 2 Methods

### 2.1 Protocol

This systematic review (SR) was performed according to the Cochrane Handbook for Systematic Reviews [26] and the guideline for Meta‐Analysis of Observational Studies in Epidemiology (MOOSE) [27]. The study is registered at the International Prospective Register of Systematic Reviews (PROSPERO) by number CRD42023439051.

### 2.2 Research Question and Hypothesis

The focused research question was formulated utilizing the population, exposure, comparison, outcomes, and study (PECOS) framework as follows [26]:

– In humans ≥ 18 years old (P = population) diagnosed with periodontitis (E = exposure) compared to those without (C = comparison), what differences in platelet status and activity (O = outcome) are reported in observational studies (S = studies)?

It was hypothesized that patients with periodontitis show differences in platelet status and activity as compared to individuals without periodontitis.

### 2.3 Search and Selection

MEDLINE‐PubMed, EMBASE, and Cochrane‐CENTRAL Library databases were used to identify all eligible studies that address the focused question, from inception up to November 2024. For details regarding the search strategy, the search terms used, and the screening and selection procedure, see Appendices S1 and S2.

The following eligibility criteria were used:

- Articles available in English and full text.
- Studies performed in humans.
- Observational studies (case–control, cross‐sectional, cohort).
- Participants 18 years or older.
- Studies evaluate a group of patients with periodontitis and a group of individuals without periodontitis.
- Studies provide a clear case definition of periodontitis, based on clinical examination evaluating probing pocket depth (PPD) in combination with clinical attachment loss (CAL) and/or radiographic bone loss.
- Studies with reported outcomes relevant to platelet status and/or activity.

### 2.4 Data Extraction and Analysis

Briefly, the selected studies were processed for data extraction and analysis. For additional details, see Appendix S1. The clinical heterogeneity was assessed according to the characteristics (age, sex, continent, smoking, and health status) and the number of participants, as well as the case definitions of periodontitis. The methodological heterogeneity factors were the study design details, the variable of interest, and the number of examined teeth per participant. The included studies were scored on the methodological qualities using the Newcastle‐Ottawa scale [28] and its adjusted version for the cross‐sectional studies [29]. A descriptive manner of data presentation was used for all studies and outcomes of interest.

Interventional studies that provided baseline data, including a group of patients with periodontitis and a control group without, were considered cross‐sectional, as the reported data exhibited observational characteristics.

As primary outcomes, the PC and mean platelet volume (MPV) were considered, and as secondary outcomes, any other biomarkers related to platelet status and activity were considered.

Meta‐analysis (MA) was conducted for the two primary outcome parameters, PC and MPV where feasible. The mean values and standard deviations were utilized and analyzed via non‐standardized mean differences along with 95% confidence intervals (CIs), applying a “random or fixed effects” model when appropriate using R Statistical Software (v4.3.0; R Core Team, April 2023). *p*‐values < 0.05 were considered as significant.

When analyzing four or more comparisons, the “random effects” model was chosen to compute the weighted average of the exposure effects across the studies. The between‐trial variance, *τ* ^2^, was estimated with the use of the restricted maximum likelihood method. If there were fewer than four studies, the “fixed effects” model was used as the estimate of inter‐trial variance is poor for analyses with low numbers of studies [26].

For MA with more than two comparisons, the 95% prediction intervals were calculated to estimate changes in PC and MPV in future studies [30].

In studies involving multiple periodontitis groups, each pairwise comparison was analyzed separately. To account for the shared control group, its data was evenly distributed across the comparisons. This approach preserved the valuable information provided by multi‐group studies and prevented issues arising from the repeated inclusion of the same control group in the analysis [26].

Heterogeneity was tested using the chi‐square test and the *I* ^2^ statistic with 95% CIs [26, 31] The chi‐square test resulting in a *p*‐value < 0.1 was an indication of significant statistical heterogeneity. To provide an estimate of the variation among the studies, an *I* ^2^ statistic of 0%–40% was deemed insignificantly inconsistent, 30%–60% as moderately heterogeneous, 50%–90% as substantially heterogeneous, and 75%–100% as considerably heterogeneous [32].

Sensitivity analyses were conducted by replicating the principal analyses after the elimination of studies based on specific aspects in the domain of clinical or methodological heterogeneity.

Meta‐regression was performed exploring the impact of the age and the sex ratio of participants on the observed mean differences, wherever feasible.

Publication bias testing for each outcome was utilized as proposed by Egger et al. [33], if ≥ 10 studies were included in the MA. The presence of asymmetry in the inverted funnel was indicative of potential publication bias [26, 34]. When no clear asymmetry in the funnel plots was observed, Egger's test was applied. The Doi plot and Luis Furuya‐Kanamori (LFK) index [35] were employed when there were < 10 studies available for evaluation. An LFK index < −1 or > 1 was indicative of negative or positive publication bias, respectively [35].

Trial sequential analysis (TSA) was employed to estimate the type Ι error risk. The required information size (RIS) and trial sequential monitoring boundaries (TSMB) for benefit or futility were computed. The RIS was determined considering a type Ι error risk of *α* = 5% and a type ΙΙ error risk of *β* = 0.20, with a statistical test power of 80%. RIS was adjusted for heterogeneity and multiple comparisons. The Lan‐DeMets version [36] of the O'Brien‐Fleming function [37] was utilized to assess the TSMBs. TSA software version 0.9.5.10 Beta (Copenhagen Trial Unit, Copenhagen, Denmark) was used [38, 39, 40, 41].

### 2.5 Grading

The quality of evidence and the strength of recommendations were evaluated by utilizing the Grading of Recommendations Assessment, Development and Evaluation (GRADE) [42, 43]. Both assessments were performed independently by two reviewers (KHLC and NDQS). In case of any discrepancies between the two reviewers, further discussion was conducted to reach a consensus. If a disagreement persisted, the final decision was made by a third reviewer (DS).

## 3 Results

### 3.1 Search and Selection Results

**FIGURE 1:**
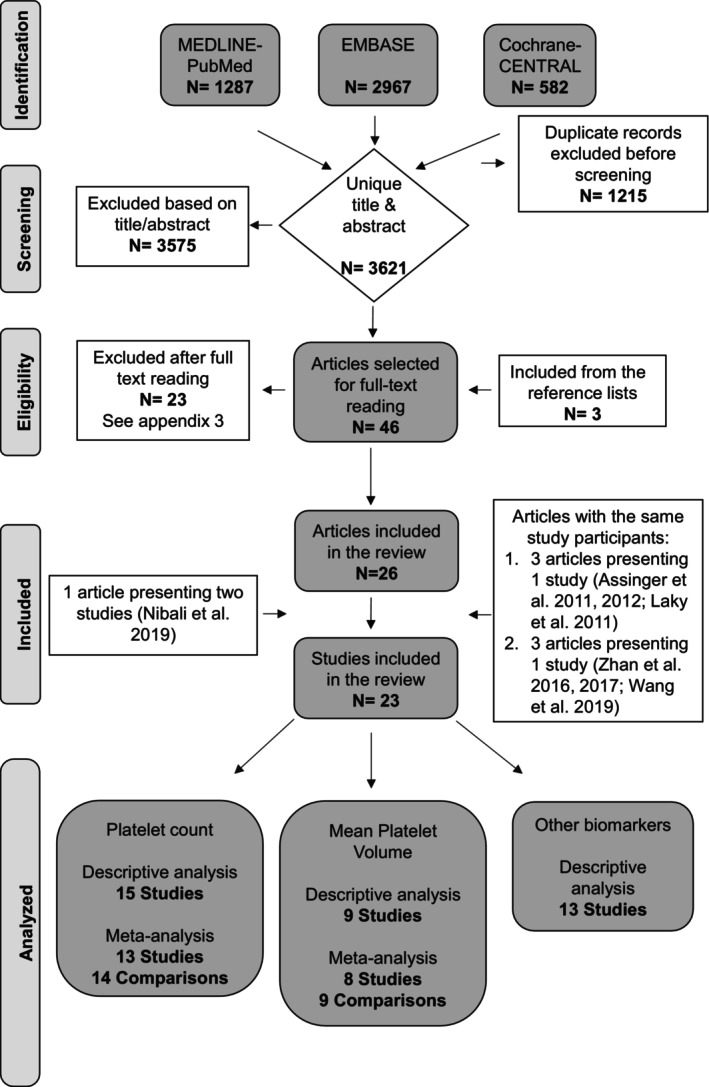
Search and selection flow chart.

The search of the MEDLINE‐PubMed, Cochrane‐CENTRAL, and EMBASE electronic databases resulted in 3621 unique records. After detailed screening, a total of 26 articles were selected. Those by Assinger et al. [4, 44] and Laky et al. [45] consisted of the same study participants. Thus, they were evaluated as a single study. The same applied to the articles by Zhan et al. [23, 24] and Wang et al. [46]. The article by Nibali et al. [47] featured two distinct studies: a main study (I) and a replication study (II). Consequently, they were treated as separate entities in the analysis. Additionally, the article by Papapanagiotou et al. [20] comprised two studies, with the second study sharing the same participant pool as the study by Nicu et al. [21]. Accordingly, the outcomes of interest from the second study in the article by Papapanagiotou et al. [20] were evaluated within the context of the study by Nicu et al. [21]. Eventually, the selected articles corresponded to 23 studies that subsequently were subjected to data extraction and analysis (Figure 1 and Appendix S3).

### 3.2 Assessment of Clinical and Methodological Heterogeneity

This SR encompasses a total of 12 630 participants across the included studies. Appendix S4 contains a comprehensive overview and information on the study designs, geographic locations, participant demographics (age and sex distribution), sample sizes, the case definitions of periodontitis, and the variables of interest. Considerable clinical and methodological heterogeneity was noted among the investigations, as outlined in detail in Appendix S5.

### 3.3 Methodological Quality Assessment

Following the Newcastle‐Ottawa scale [28, 29], 14 studies (11 cross‐sectional and 3 case–control) were rated as low risk of bias [4, 20, 21, 23, 24, 44, 45, 46, 47, 48, 49, 50, 51, 52, 53, 54, 55] and 9 cross‐sectional studies as moderate risk of bias [22, 56, 57, 58, 59, 60, 61, 62, 63] (Appendix S6A,B).

### 3.4 Descriptive Analysis

Tables 1 and 2 presents a summary of the statistical significance levels for differences in variables relevant to platelet status and activity. In total, 22 variables of interest were assessed across the included studies to discern differences in platelet status between patients with periodontitis and individuals without. Appendix S7 provides an overview of the results and original authors' conclusions derived from the selected investigations.

**TABLE 1:** Descriptive summary of statistical significance levels of the difference regarding platelet count and mean platelet volume.

Variable of interest	Outcomes (for interpretation of the colors see footnote)
	Sample size per group
	Number of studies (cited articles)
Platelet count	Control: *n* = 148 Periodontitis, *n* = 149 4 studies [21, 56, 58, 60]
	Control: *n* = 6247 Periodontitis, *n* = 5647 11 studies [23, 24, 46, 47, 48, 49, 50, 52, 53, 54, 55, 57]^a^, ^b^
Mean platelet volume	Control: *n* = 82 Periodontitis, *n* = 107 2 studies [55, 61]
	Control: *n* = 440 Periodontitis, *n* = 446 4 studies [23, 24, 46, 47, 52, 63]^a^
	Control: *n* = 301 Periodontitis, *n* = 327 3 studies [47, 49, 56]

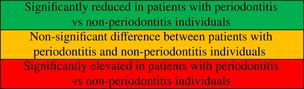

^a^ Articles [23, 24, 46] consisted of the same study participants, and they were evaluated as a single study.

^b^ Article [47] featured two distinct studies which were treated as separate entities in the analysis.

**TABLE 2:** Descriptive summary of statistical significance levels of the difference regarding other biomarkers relating to platelets status and activity.

Variable of interest	Outcomes (for interpretation of the colors see footnote)
	Sample size per group
	Number of studies (cited articles)
Platelet large cell ratio	Control: *n* = 184 Periodontitis, *n* = 269 2 studies [23, 24, 46, 52]^a^
Intracellular VASP phosphorylation	Control: *n* = 19 Periodontitis, *n* = 26 1 study [4, 44, 45]^b^
Plateletcrit	Control: *n* = 82 Periodontitis, *n* = 107 2 studies [55, 61]
Platelet activation, coagulation signals and Rho GTPase effectors expression	Control: *n* = 11 Periodontitis, *n* = 16 1 study [59]
PAC1 binding ability	Control: *n* = 18 Periodontitis, *n* = 19 1 study [21]
Intraplatelet L‐arginine‐NO‐cGMP pathway & oxidant‐antioxidant balance	Control: *n* = 8 Periodontitis, *n* = 8 1 study [62]
Platelet P‐selectin surface expression	Control: *n* = 19 Periodontitis, *n* = 26 1 study [4, 44, 45]^b^
Platelet CD40L surface expression	Control: *n* = 19 Periodontitis, *n* = 26 1 study [4, 44, 45]^b^
GPIIb/IIIa activation	Control: *n* = 19 Periodontitis, *n* = 26 1 study [4, 44, 45]^b^
Phosphatidylserine levels	Control: *n* = 19 Periodontitis, *n* = 26 1 study [4, 44, 45]^b^
Platelet‐monocyte complexes	Control: *n* = 18 Periodontitis, *n* = 19 1 study [21]
Platelet–neutrophil complexes	Control: *n* = 18 Periodontitis, *n* = 19 1 study [21]
PAC1 expression	Control: *n* = 18 Periodontitis, *n* = 19 1 study [21]
CD63 expression	Control: *n* = 18 Periodontitis, *n* = 19 1 study [21]
Platelet morphology	Fewer small form, more spider form Control: *n* = 40 Periodontitis, *n* = 40 1 study [60]
	Big form Control: *n* = 40 Periodontitis, *n* = 40 1 study [60]
sP‐selectin expression	Control: *n* = 54 Periodontitis, *n* = 111 2 studies [4, 20, 44, 45]^b^
	Positive correlation with CAL Control: *n* = 40 Periodontitis, *n* = 40 1 study [60]
	Control: *n* = 38 Periodontitis, *n* = 44 2 studies [21, 51]
sCD40L expression	Control: *n* = 20 Periodontitis, *n* = 25 1 study [51]
	Control: *n* = 72 Periodontitis, *n* = 130 3 studies [4, 20, 21, 44, 45]^b^
Platelet aggregation	Control: *n* = 40 Periodontitis, *n* = 40 1 study [60]
	After platelets stimulation with fibrillar collagen Control: *n* = 8 Periodontitis, *n* = 8 1 study [62]
PF4	Circulating Control: *n* = 7 Periodontitis, *n* = 4 1 study [22]
	Intraplatelet concentrations Control: *n* = 7 Periodontitis, *n* = 4 1 study [22]
Platelet distribution width	Control: *n* = 25 Periodontitis, *n* = 50 1 study [61]
	Control: *n* = 139 Periodontitis, *n* = 224 1 study [23, 24, 46]^a^

*Note:* For interpretation of the colors see Table 1. Abbreviations: CAL, Clinical Attachment Levels; CD40L, Cluster of Differentiation 40 Ligand; CD63, Cluster of Differentiation 63 antigen; cGMP, cyclic Guanosine Monophosphate; GPIIb/IIIa, Glycoprotein IIb/IIIa (also known as integrin αIIbβ3); GTP, Guanosine Triphosphate; NO, Nitric Oxide; PAC‐1, first Procaspase Activating Compound; PF4, Platelet Factor 4; Rho, Ras homolog; sCD40L, soluble Cluster of Differentiation 40 Ligand; sP‐selectin, soluble P‐selectin; VASP, Vasodilator‐Stimulated Phosphoprotein.

^a^ Articles [23, 24, 46] consisted of the same study participants, and they were evaluated as a single study.

^b^ Articles [4, 44, 45] consisted of the same study participants, and they were evaluated as a single study.

### 3.5 Primary Outcomes of Interest

Among the 15 studies that evaluated the PC, eleven studies did not find significant differences between individuals with periodontitis and those without [23, 24, 46, 47, 48, 49, 50, 52, 53, 54, 55, 57] and four indicated significantly higher platelet numbers in patients with periodontitis compared to non‐periodontitis counterparts [21, 56, 58, 60] (Table 1). Among the eleven studies that reported no significant differences in PC between the two groups, seven [23, 24, 46, 47, 48, 52, 53, 55, 57] exhibited a non‐significant numerical trend toward higher PC levels in patients with periodontitis. Moreover, the study by Nibali et al. II [47], which in general did not find a significant difference, observed a significant elevation of platelets in the sub‐analysis on male patients formerly diagnosed with aggressive periodontitis in comparison to the control group.

Regarding MPV, four studies [23, 24, 46, 47, 52, 63] ascertained significantly lower levels in patients with periodontitis, two studies [55, 61] noted significantly higher levels in patients with periodontitis, and three studies detected no significant difference between the two groups [47, 49, 56] (Table 1).

### 3.6 Secondary Outcomes of Interest

All studies examining the platelet large cell ratio and intracellular vasodilator‐stimulated phosphoprotein phosphorylation revealed significantly lower levels in patients with periodontitis [4, 23, 24, 44, 45, 46, 52] (Table 2).

Meanwhile, studies evaluating plateletcrit, first procaspase activating compound (PAC1) binding ability, the intraplatelet L‐arginine‐nitric oxide‐cyclic guanosine monophosphate pathway, and the oxidant–antioxidant balance, the activation and coagulation signals levels, and the expression of genes for ras homolog guanosine triphosphatase effectors identified significantly higher levels in patients with periodontitis [21, 55, 59, 61, 62] (Table 2).

Additionally, the studies investigating the platelets surface expression of P‐selectin and cluster of differentiation 40 ligand (CD40L), the platelets‐monocytes and platelets‐neutrophils formation complexes, the glycoprotein IIb/IIIa activation and phosphatidylserine levels, and the expressions of PAC1 and cluster of differentiation 63 antigen did not observe significant differences between individuals with periodontitis and those without [4, 21, 44, 45] (Table 2).

For the remaining six variables of interest (platelet distribution width and morphology, soluble P‐selectin and CD40L, platelets aggregation, and platelet factor 4) some investigations showed significant differences in favor of the periodontitis group, while others found no significant differences between the groups [4, 20, 21, 22, 23, 24, 44, 45, 46, 51, 60, 61, 62] (Table 2).

### 3.7 Meta‐Analysis and TSA

Thirteen included studies [21, 23, 24, 46, 47, 48, 50, 52, 53, 55, 56, 57, 58, 60] presenting 14 comparisons were proceeded with for the MA and TSA on PC. The studies by Temelli et al. [49] and Zhao et al. [54] were omitted due to the provision of only median data. Additionally, the two studies conducted by Nibali et al. [47] required a recalculation for both groups because they initially segregated data by sex in each group, which was performed with the use of sample.decomp in the utilities package of R. Table 3 summarizes the outcomes of the MA and the sub‐analyses based on periodontitis case definition, risk of bias, study design, and smoking. In the sub‐analysis based on periodontitis case definition, the former diagnosis of aggressive periodontitis [64] was redefined as molar/incisor or generalized stage 3–4, grade C, due to rapid progression and/or early onset periodontitis, in accordance with the 2017 World Workshop on the classification of periodontal and peri‐implant diseases and conditions [65]. This is aligned with the periodontitis case definition criteria applied in the original studies included in this sub‐analysis [23, 24, 46, 47, 50]. Appendices S8–S12 present the corresponding forest plots.

**TABLE 3:** Overview of overall meta‐analysis for platelet count and sub‐analyses based on periodontitis case definition, risk of bias, study design, and smoking.

	Included studies	Effect sizes					Heterogeneity		Funnel plot see Appendix	For details see Appendix
	Comparisons	MD (×10^9^/L)	Model	95% CI	*p*	95% Prediction interval	*I* ^2^ value [95% CI]	*p*		
Overall	13 studies [21, 23, 24, 46, 47, 48, 50, 52, 53, 55, 56, 57, 58, 60] 14 comparisons	23.55	Random	[7.68; 39.43]	< 0.01	[−39.33; 86.44]	84% [75%; 90%]	< 0.01	S13	S8
Periodontitis case definition										
Molar/incisor or generalized stage 3–4, Grade C, due to rapid progression and/or early onset	[23, 24, 46, 47, 50]	1.79	Random	[−5.06; 8.65]	0.61	NA	0% [0%; 85%]	0.81	S13	S9A
Non‐molar/incisor or generalized stage 3–4, Grade C, due to rapid progression and/or early onset	[21, 47, 48, 52, 53, 55, 56, 57, 58, 60]	34.53	Random	[14.57; 54.48]	< 0.01	NA	86% [76%; 92%]	< 0.01	S13	S9B
Risk of bias										
Low	[21, 23, 24, 46, 47, 48, 50, 52, 53]	5.64	Random	[0.72; 10.57]	0.02	NA	26% [0%; 65%]	0.20	S13	S10A
Moderate	[55, 56, 57, 58, 60]	71.55	Random	[56.50; 86.59]	< 0.01	NA	0% [0%; 85%]	0.43	S13	S10B
Study design										
Case–control	[21, 23, 24, 46, 47, 50, 52, 53, 55, 57, 58, 60]	2.06	Fixed	[−7.13; 11.25]	0.66	NA	0% [0%; 90%]	0.50	S13	S11A
Cross‐sectional	[48, 56]	29.96	Random	[10.90; 49.02]	< 0.01	NA	87% [78%; 92%]	< 0.01	S13	S11B
Smoking										
Including smokers	[21, 47]	2.36	Random	[−5.36; 10.09]	0.55	NA	47% [0%; 82%]	0.13	S13	S12A
Excluding smokers	[23, 24, 46, 48, 50, 52, 53, 55, 56, 57, 58, 60]	30.88	Random	[10.06; 51.70]	< 0.01	NA	86% [77%; 92%]	< 0.01	S13	S12B

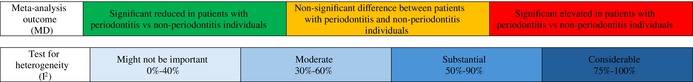

*Note:* As a guideline, to assess the potential magnitude of incosistency between studies, an *I* ^2^ statistic of 0%–40% may represent unimportant levels of heterogeneity, 30%–60% moderate heterogeneity, 50%–90% substantial heterogeneity, and > 75% considerable heterogeneity.

Abbreviations: CI, confidence interval; MD, mean difference; NA, not applicable.

**FIGURE 2:**
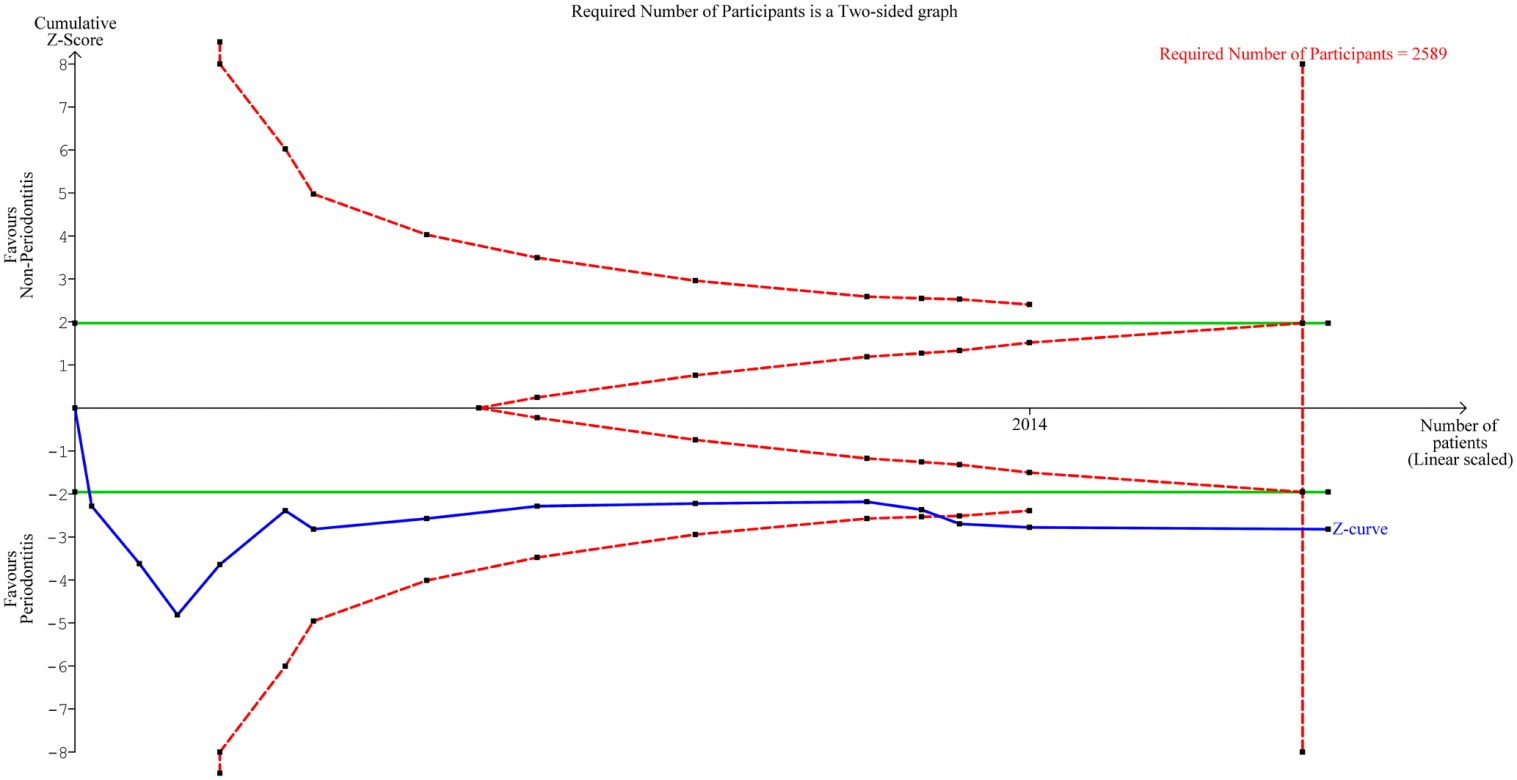
Trial sequential analysis (TSA) of the selected studies for the platelet count. The cumulative blue Z‐curve was constructed with each cumulative Z‐value calculated after including a new trial according to the publication date. The green lines represent the traditional boundary. The oblique red dashed lines represent the trial sequential monitoring boundaries and the futility boundaries. The vertical red dashed line represents the estimated heterogeneity‐adjusted required information size, and the number of participants for the meta‐analysis sample size.

The overall MA on PC showed that patients with periodontitis (*n* = 1376) had a significantly higher number of platelets compared to individuals without periodontitis (*n* = 1268) (Mean Difference (MD) = 23.55 ×10^9^/L; 95% CI [7.68; 39.43] *p* < 0.01) with considerable statistical heterogeneity (*I* ^2^ = 84%). The 95% prediction interval (PI) for the estimation of PC in future studies disclosed a range of −39.33; 86.44 ×10^9^/L (Table 3, Appendix S8). The Z‐curve in the TSA crossed the monitoring boundaries and reached the required number of participants (*n* = 2589) (Figure 2). The funnel plot disclosed asymmetry indicative of potential publication bias, which was confirmed by the Egger's test (*p* = 0.01) (Appendix S13).

All sub‐analyses indicated higher PC in patients with periodontitis compared to individuals without. However, no statistically significant differences were observed within those involving patients with molar/incisor or generalized stage 3–4, grade C due to rapid progression and/or early onset periodontitis, case–control studies, or smokers. The heterogeneity might not be important in half of the sub‐analyses. However, it was categorized as considerable in sub‐analyses of studies including patients with non‐molar/incisor or generalized stage 3–4, grade C due to rapid progression and/or early onset periodontitis, or with cross‐sectional design, or those excluding smokers and moderate in those including smokers (For details see Table 3, Appendices S9–S12). To explore the impact of age and sex ratio on the observed heterogeneity, meta‐regressions were conducted, with the exclusion of one study lacking mean or median age and sex ratio data [60]. The analysis did not reveal a significant age‐related (*p* = 0.28) or sex‐related effect (*p* = 0.18) (Appendices S14 and S15).

The MA for MPV included eight studies [23, 24, 46, 47, 52, 55, 56, 61, 63], presenting 9 comparisons. The study by Temeli et al. [49] was excluded as for PC. The results from the two studies conducted by Nibali et al. [47] were recalculated in a manner analogous to the results for PC in the same studies, using the sample.decomp in the utilities package of R. Similarly, in the study by Mutthineni et al. [61], data from patients with periodontitis were reanalyzed and consolidated into a single group, combining the results from the moderate and severe periodontitis groups initially reported [47]. A similar recalculation was performed for the results in the study by Dolma et al. [63], which were initially categorized into periodontitis smokers, periodontitis non‐smokers, control smokers, and control non‐smokers groups. The data for the two periodontitis groups were combined into one group, as were the data for the two control groups. Table 4 summarizes the MA results for MPV and corresponding sub‐analyses, categorized by periodontitis case definition, risk of bias, study design, and smoking. In the sub‐analysis based on periodontitis case definition, the former diagnosis of aggressive periodontitis was reclassified in line with the criteria applied in the MA for PC. For the forest plots, see Appendices S16–S20.

**TABLE 4:** Overview of overall meta‐analysis for mean platelet volume and sub‐analyses based on periodontitis case definition, risk of bias, study design, and smoking.

	Included studies	Effect sizes					Heterogeneity		Doi plot see Appendix	For details see Appendix
	Comparisons	MD (fL)	Model	95% CI	*p*	95% Prediction interval	*I* ^2^ value [95% CI]	*p*		
Overall	8 studies [23, 24, 46, 47, 52, 55, 56, 61, 63] 9 comparisons	0.16	Random	[−0.49; 0.82]	0.62	[−2.31; 2.64]	96% [94%; 97%]	< 0.01	S21	S16
Periodontitis case definition										
Molar/incisor or generalized stage 3–4, Grade C, due to rapid progression and/or early onset	[23, 24, 46, 47]	−0.19	Fixed	[−0.32; −0.06]	< 0.01	NA	77% [26%; 93%]	0.01	S21	S17A
Non‐molar/incisor or generalized stage 3–4, Grade C, due to rapid progression and/or early onset	[47, 52, 55, 56, 61, 63]	0.35	Random	[−0.64; 1.34]	0.48	NA	97% [96%; 98%]	< 0.01	S21	S17B
Risk of bias										
Low	[23, 24, 46, 47, 52]	−0.05	Random	[−0.36; 0.26]	0.75	NA	84% [67%; 92%]	< 0.01	S21	S18A
Moderate	[56, 61, 63]	−0.11	Fixed	[−0.31; 0.09]	0.27	NA	99% [98%; 99%]	< 0.01	S21	S18B
Study design										
Case–control	[23, 24]	NA	NA	NA	NA	NA	NA	NA	S21	NA
Cross‐sectional	[56, 61, 63]	0.22	Random	[−0.52; 0.96]	0.56	NA	96% [95%; 98%]	< 0.01	S21	S19
Smoking										
Including smokers	[47, 63]	−0.21	Random	[−0.69; 0.27]	0.39	NA	94% [88%; 97%]	< 0.01	S21	S20A
Excluding smokers	[23, 24, 46, 52, 55, 56, 61]	0.48	Random	[−0.65; 1.60]	0.40	NA	97% [95%; 98%]	< 0.01	S21	S20B

*Note:* For interpretation of the colors see table 3.

Abbreviations: CI, confidence interval; MD, mean difference; NA, not applicable.

**FIGURE 3:**
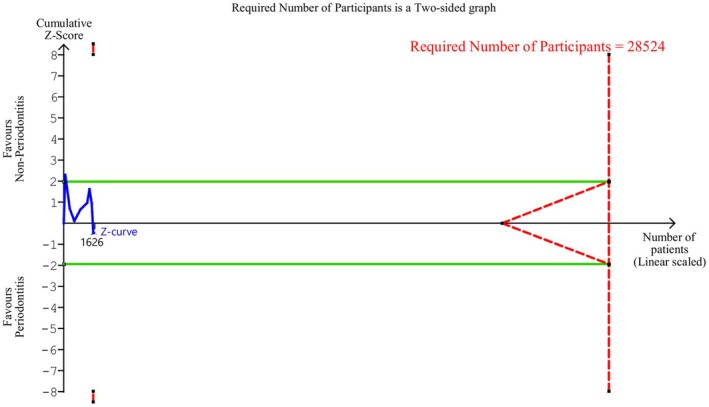
Trial sequential analysis (TSA) of the selected studies for the mean platelet volume. *Note:* For interpretation of the graph see Figure 2.

In the overall meta‐analysis for MPV, no significant difference was observed between the periodontitis group (*n* = 839) and the non‐periodontitis group (*n* = 787) (MD = 0.16 fL; 95% CI [−0.49, 0.82]; *p* = 0.62). Considerable heterogeneity was present (*I* ^2^ = 96%), with a 95% prediction interval ranging from −2.31 to 2.64 fL (Table 4, Appendix S16). Figure 3 shows the corresponding TSA; the Z‐curve did not cross below the futility boundaries and did not reach the required number of participants (*n* = 28 524). The Doi plot demonstrated minor asymmetry, and the LFK index was 1.69, suggesting publication bias (Appendix S21).

All sub‐analyses exhibited no statistically significant differences in MPV between the two groups and considerable heterogeneity, except for the analysis of studies including patients with molar/incisor or generalized stage 3–4, grade C due to rapid progression and/or early onset periodontitis, which revealed a statistically significant lower MPV in the periodontitis group and substantial heterogeneity (Table 4, Appendices S17–S20).

### 3.8 Evidence Profile

Table 5 presents a summary of the diverse factors utilized for rating and evaluating the certainty of the quality of the evidence and the strength of the recommendations in accordance with GRADE [42, 43].

**TABLE 5:** GRADE evidence profile for the differences in platelet count, mean platelet volume, and other biomarkers among patients with periodontitis as compared to non‐periodontitis individuals.

Summary of the estimated evidence profile				
Determinants of quality	Platelet count	Mean platelet volume	Other biomarkers	Sources
Study design	Observational studies	Observational studies	Observational studies	Appendix S4
# studies # comparisons in MA	# 15 # 14	# 9 # 9	# 13 Not applicable	Appendices S8, S16, Figure 1, Tables 1, 2, 3, 4
Risk of bias	Low to moderate	Low to moderate	Low to moderate	Appendix S6A,B
Consistency	Rather inconsistent	Rather inconsistent	Inconsistent	Appendices S7–S12, S16–S20, Tables 1, 2, 3, 4
Directness	Rather indirect	Rather indirect	Rather indirect	Appendices S4, S8–S12, S16–S20, Tables 1, 2, 3, 4
Precision	Precise	Rather precise	Rather imprecise	Appendices S8–S12, S16–S20, Figures 2 and 3, Tables 3 and 4
Reporting bias	Suspected	Suspected	Possible	Appendices S13, S21
Magnitude of the effect	Small	Non‐significant	Unclear	Appendices S8–S12, S16–S20, Tables 3 and 4 and [31, 32]
Strength of the recommendation based on the quality and body of evidence	Moderate	Low	Very low	
	⊕⊕⊕	⊕⊕	⊕	Figures 2 and 3, Tables 1 and 2
Direction of recommendation	The certainty is moderate for slightly higher platelet count in patients with periodontitis compared to individuals without periodontitis and low for a non‐significant difference in mean platelet volume between the two groups. No recommendation can be made for the other biomarkers, based on the current available evidence.			

The certainty is moderate for slightly higher PC in patients with periodontitis compared to individuals without periodontitis and low for no difference in MPV between the two groups. No recommendation can be made for the other biomarkers, based on the currently available evidence (Table 5).

## 4 Discussion

This SR aimed to summarize, assess, and synthesize the available evidence regarding the platelets' status and activity among individuals with periodontitis, based on the hypothesis that these patients could show differences in the status and activity of platelets compared to individuals without periodontitis.

### 4.1 Summary and Explanation of the Findings

Twenty‐two variables pertinent to platelet activity were evaluated across the included studies, with PC and MPV being the most frequently assessed. Platelets are complex, multi‐functional blood cells whose activation is crucial for hemostasis, host defense, and various pathological conditions, including CVDs. Their mobilization is characterized by modifications in their number, volume, shape, surface adhesion molecules expression, and constituents' secretion [1, 2, 3, 8]. Consequently, varied methods and biomarkers have been employed to assess their status, with no single test being universally recognized as the gold standard [8]. Nonetheless, PC and MPV are commonly utilized as predictors in CVD's and autoimmune diseases as well [66, 67, 68].

Our findings demonstrated a small yet statistically significantly higher number of platelets in patients with periodontitis compared to controls. This MA finding was corroborated by TSA, with the Z‐curve crossing the monitoring boundaries and reaching the required number of participants, thus excluding the type I error and indicating the MA results as conclusive. On the other hand, MPV was not significantly different between the two groups. TSA indicated that this MA was inconclusive, as the Z‐curve did not cross the futility boundaries nor obtain the required number of participants. Respecting other biomarkers used to evaluate shifts in platelet status and activity, the findings were heterogeneous and limited due to the small number of studies available for each biomarker [4, 20, 21, 22, 23, 24, 44, 45, 46, 51, 55, 59, 60, 61, 62].

The normal PC range is 150–400 ×10^9^/L [69]. Previous research has demonstrated that PC levels exceeding 251 ×10^9^/L, although within the normal range, are associated with an elevated risk of developing CVDs, particularly in men and individuals under 65 years of age [70, 71]. In the present MA, the mean PC in patients with periodontitis was 262.89 ×10^9^/L and significantly higher than that observed in individuals without periodontitis (MD = 23.55 ×10^9^/L). This finding highlights the potential pro‐thrombotic state associated with periodontitis [72].

Further, both meta‐analyses for PC and MPV revealed considerable heterogeneity, signifying variance in the exposure effects among included studies and wide prediction intervals prognosticating possible lower levels of PC and MPV in patients with periodontitis compared to control individuals. Collaboratively, these observations may reflect the existence of patients with periodontitis subpopulations with less prominent differences in platelet status [30, 73].

Periodontitis is linked to an increased incidence of bacteremia with oral pathogens and higher levels of inflammatory biomarkers in the bloodstream in comparison to non‐periodontitis individuals [15, 16, 17, 18, 19, 74]. These conditions can result in an increase of platelet numbers and their activation, with variable platelet responses against different bacteria [21, 75]. Activated platelets are larger and able to form complexes with leukocytes, which can aid in bacterial clearance [21]. The diminished size of platelets observed in patients with molar/incisor or generalized stage 3–4, grade C due to rapid progression and/or early onset periodontitis may be attributed to the increased consumption of larger, activated platelets at the sites of inflammation due to their aggregation with leukocytes [24, 46, 76, 77, 78]. Similar observations have been documented in other inflammatory diseases like rheumatoid arthritis, ulcerative colitis, systemic lupus erythematosus, and ankylosing spondylarthritis [79, 80, 81, 82]. In addition, the meta‐regression analyses which explored the impact of age and sex ratio in the observed heterogeneity of the MA for PC exhibited a trend toward a smaller difference in PC between patients with periodontitis and controls among younger individuals and females. Thus, these results may underscore the potential diverse role of platelets in various clinical manifestations of periodontitis, contingent on the severity, extent, acute forms, and the oral microbiota characteristics of the disease. However, this hypothesis should be further investigated in future studies.

Smoking has been shown to influence platelet status [83, 84, 85]. The sub‐analyses of PC and MPV based on participants' smoking status displayed that differences in the number and volume of platelets among patients with periodontitis in studies including smokers were less pronounced compared to those in studies excluding smokers. This observation may be elucidated by variations in sex ratios across studies, as smoking could affect platelets differently in males and females [84].

Only three studies involving patients with comorbidities were incorporated in this SR [49, 54, 59]. However, all were excluded from the MA. The studies conducted by Temelli et al. [49] and Zhao et al. [54] presented median values for PC [49, 54] and MPV [49]. The study by Lee et al. [59] assessed only the transcriptional profiles of platelets. Consequently, drawing conclusions regarding the potential synergistic interaction between periodontitis and other diseases on platelet status was not possible.

### 4.2 Results in Relation to Previous Evidence

To the best of our knowledge, this is the first SR and MA investigating the platelet status and activity among patients with periodontitis. Previously, Botelho et al. [86] systematically reviewed the evidence in circulating blood cell profile alterations in patients with periodontitis, including PC and MPV. Their analysis revealed higher, but statistically non‐significant, PC in patients with periodontitis compared to the control group, whereas patients formerly diagnosed with aggressive periodontitis showed a lower, but statistically non‐significant, number of platelets as compared to individuals without periodontitis. With respect to MPV, the same study disclosed that it was lower, although statistically non‐significant, in patients with periodontitis. However, this difference reached statistical significance in patients formerly diagnosed with aggressive periodontitis, which is in accordance with our study, while patients formerly diagnosed with chronic periodontitis exhibited statistically significant higher MPV. Discrepancies between our findings and those of Botelho et al. [86] may be ascribed to differences in the studies' inclusion criteria, particularly concerning the definition of periodontitis cases based solely on PPD [87, 88], and the inclusion of participants aged < 18 years old [89]. Moreover, in our quantitative analysis, we excluded studies that used median values for PC or MPV [49], as the calculation of means and standard deviations from medians remains a topic of debate [90]. Further, our study encompassed more recent studies published since 2021 and incorporated them into our sample [48, 53, 56, 61].

### 4.3 Strengths and Limitations

A comprehensive and reproducible methodology was applied to offer a thorough overview and analysis of the existing evidence concerning differences in platelet status between patients with periodontitis and individuals without. Our stringent inclusion criteria for defining periodontitis cases aimed to minimize the frequently observed variability in this regard. Additionally, the integration of TSA and prediction intervals strengthened the MA and provided additional support for interpreting the results and making recommendations regarding future studies.

Nevertheless, this review acknowledges certain limitations that warrant discussion. Our data exclusively stem from observational studies, which restricts our ability to draw conclusions regarding the potential causal effects of periodontitis on the platelet status. The inclusion of interventional studies could offer valuable insights into platelet status in patients with periodontitis. However, such studies focus on assessing changes in platelet status and activity before and after periodontal treatment or in comparison to a control group receiving no treatment or a control intervention. As a result, their research objectives and hypotheses differ from those explored in our study.

The eligibility criteria of the study could be enhanced by additional details regarding the periodontal status of non‐periodontitis individuals. However, all included studies explicitly reported in their results that non‐periodontitis individuals exhibited no clinical or radiographic signs indicative of periodontitis.

Additionally, the studies incorporated in the review presenting data for the MPV and the secondary outcomes might not offer a representative depiction of the broader population, primarily due to the predominance of limited sample sizes within them. Thus, it is emphasized as the necessity for future studies on these biomarkers with a larger number of participants.

Moreover, the considerable heterogeneity observed in both MA requires caution when interpreting the study results. This heterogeneity may be attributed to variations in sample sizes of the studies, their methodological quality, the applied periodontitis definition criteria, the measurement methods [55] and confounding factors such as smoking [84, 85], stress [91], the physical activity levels [92], genetic characteristics [55], and the oral microbiological composition of the participants [21, 75].

Also, signs of publication bias were detected in both PC and MPV analyses, which may be related to the high heterogeneity. It is noteworthy that the study by Mutthineni et al. [61] could potentially influence the observed asymmetry in the Doi plot.

## 5 Conclusions

### 5.1 Implications for Clinical Practice

Within its limitations, the current review indicates the presence of moderate certainty for slightly higher PC in patients with periodontitis compared to individuals without periodontitis, and low certainty for no difference in MPV between the two groups. In the context of other representative platelet activation biomarkers, varied differences were observed that do not provide robust evidence to claim a clear difference between the two groups.

Platelet alterations may reflect the local and systemic inflammatory responses within patients with periodontitis. The added knowledge may enhance our understanding of the link between periodontitis and cardiovascular diseases.

### 5.2 Implications for Research

Although a possible alteration of platelets in patients with periodontitis is biologically plausible, up to now, epidemiological studies have failed to provide data with sufficient quantity and quality to draw conclusions based on solid scientific evidence. Future investigations necessitate larger sample sizes, broader evaluations of biomarkers relevant to platelet activation, and longitudinal study designs to elucidate potential causative relationships between periodontitis and modifications in the platelet status. These studies should adhere to widely accepted periodontitis case definition criteria [65, 93] and should account for essential adjustments to address confounding variables, such as comorbidities, smoking and stress statuses, and the physical activity levels of the participants that might introduce bias in their outcomes.

## Author Contributions

Dimitris Sokos: contributed to conception, design, search, selection, analysis, interpretation, and drafted the manuscript. Marja L. Laine: contributed to conception, design, interpretation, and critically revised the manuscript. Elena A. Nicu: contributed to conception, interpretation, and critically revised the manuscript. Kelly Hiu Lam Chung: contributed to the search, selection, analysis, and critically revised the manuscript. Ni‐ni Dong Qing Sluijk: contributed to the search, selection, analysis, and critically revised the manuscript. Dagmar Else Slot: contributed to the analysis, interpretation and critically revised the manuscript. Sergio Bizzarro: contributed to the interpretation and critically revised the manuscript. All authors gave final approval and agreed to be accountable for all aspects of the work ensuring integrity and accuracy.

## Ethics Statement

The local ACTA ethical committee approved the study (2023‐86248).

## Conflicts of Interest

The authors declare no conflicts of interest.

## Supporting information


Appendix S1.–S21.

## Data Availability

Data were derived from resources available in original papers that are published in the public domain.
