# Supplementary material for: Periodontitis and Platelets Status: A Systematic Review With Meta‐Analysis and Trial Sequential Analysis
Source: J Periodontal Res. 2025 Mar 11;60(9):872–88. doi: 10.1111/jre.13398 (PMC12517253; doi:10.1111/jre.13398)

**Appendices**

**Periodontitis and platelets status:**

**a systematic review with meta-analysis and trial sequential analysis**

**Dimitris Sokos** (ORCID: 0000-0002-5609-0864)

**Marja L. Laine** (ORCID: 0000-0001-6052-041X)

**Elena A. Nicu** (ORCID: 0000-0002-2443-0019)

**Kelly Hiu Lam Chung**

**Ni-ni Dong Qing Sluijk**

**Dagmar Else Slot** (ORCID: 0000-0001-7234-0037)

**Sergio Bizzarro** (ORCID: 0000-0002-5228-5998)

**Appendix 1**

**Search strategy**

The search was developed collaboratively by three reviewers (DS, KHLC, NDQS). Appendix 2A-C provide the complete search strategy and search terms employed for each corresponding database.

**Screening and selection**

The screening and selection were performed by two blind reviewers (KHLC and NDQS). After excluding duplicates, the remaining records were first screened based on their titles and abstracts, using Rayyan (1). Then, the selected articles that potentially met the inclusion criteria or for which the title and abstract provided insufficient information to make a clear decision were further assessed via full-text reading. Furthermore, the reference lists of the articles selected for full-text reading in this review were hand-searched to identify additional potentially relevant articles.

Any disagreement between the two reviewers was discussed and a consensus was tried to be reached. When the reviewers could not agree a third reviewer (DS) was consulted to make the ultimate decision. In cases where the same study population was assessed across multiple articles, it was treated as a single study. Conversely, if a single article reported multiple studies and provided all requisite data, each study was processed individually. Subsequently, studies that fulfilled all eligibility criteria were determined and included in the systematic review for data extraction and methodological quality assessment. In case of studies with insufficient or unclear data, the corresponding authors were contacted via e-mail to inquire whether additional data could be provided.

**Data extraction**

Two reviewers (KHLC and NDQS) extracted independently the data from the selected studies by means of a custom-designed standardized data extraction form. Disagreement between the two reviewers was resolved through discussion and consensus. If disagreement persisted, a third reviewer (DS) was consulted; this judgment was decisive.

From the eligible studies, details on: 1) the first author and the year of publication, 2) the location of the study, 3) the design of the study, 4) the age and the sex of the participants, 5) the sample size, 6) the periodontitis case definition, 7) the variable of interest, 8) the outcomes and 9) the conclusions of the original authors were extracted.

**Methodological quality assessment**

Studies with at least seven stars were categorized as having a low risk of bias, those with five or six stars as moderate risk of bias and studies with fewer than five stars as high risk of bias. The risk of bias assessment was performed independently by two blind reviewers (KHLC and NDQS). Disagreement between the two reviewers was resolved through discussion and consensus. If disagreement persisted, a third reviewer (DS) was consulted; this judgment was decisive.

**Reference Appendix 1:**

1. Ouzzani M, Hammady H, Fedorowicz Z, Elmagarmid A. Rayyan—a web and mobile app for systematic reviews. Syst Rev. 2016;5(1):210.

**Appendix 2**

A. Search terms MEDLINE-PubMed

| Periodontitis AND Platelets |
| --- |
| ((periodontitis[MeSH Terms]) OR (periodontal disease[MeSH Terms])) OR (periodontitis[Title/Abstract])) OR (periodontal disease[Title/Abstract])) OR (periodonti*[Title/Abstract])) OR (pericementi*[Title/Abstract])) OR (parodontos*[Title/Abstract])) OR (pyorrhea alveolaris[Title/Abstract]))  AND  ((blood platelets[MeSH Terms]) OR (platelets[MeSH Terms])) OR (blood platelet*[Title/Abstract])) OR (platelet*[Title/Abstract])) OR (thrombocyt*[Title/Abstract])) OR (platelet function[Title/Abstract])) OR (platelet aggregation[Title/Abstract])) OR (platelet count[Title/Abstract])) OR (mean platelet volume[Title/Abstract])) OR (platelet activation[Title/Abstract])) |

B. Search terms EMBASE

| Periodontitis AND Platelets |
| --- |
| exp periodontitis/ OR exp periodontal disease OR periodontitis.ab,kf,ti. OR periodontal disease.ab,kf,ti. OR "periodonti*".ab,kf,ti. OR "pericementi*".ab,kf,ti. OR "parodontos*".ab,kf,ti. OR pyorrhea alveolaris.ab,kf,ti.  AND  exp thrombocyte/ OR "blood platelet*".ab,kf,ti. OR "platelet*".ab,kf,ti. OR "thrombocyt*".ab,kf,ti. OR platelet function.ab,kf,ti. OR platelet aggregation.ab,kf,ti. OR platelet count.ab,kf,ti. OR mean platelet volume.ab,kf,ti. OR platelet activation.ab,kf,ti. |

C. Search terms Cochrane-CENTRAL

| Periodontitis AND Platelets |
| --- |
| MeSH descriptor: [Periodontitis] OR [Periodontal Diseases] OR (periodontitis):ti,ab,kw OR (periodontal disease):ti,ab,kw OR (periodonti*):ti,ab,kw OR (pericementi*):ti,ab,kw OR (parodontos*):ti,ab,kw OR (pyorrhea alveolaris):ti,ab,kw  AND  MeSH descriptor: [Blood Platelets] OR (blood platelet*):ti,ab,kw OR (platelet*):ti,ab,kw OR (thrombocyt*):ti,ab,kw OR (platelet function):ti,ab,kw OR (platelet aggregation):ti,ab,kw OR (platelet count):ti,ab,kw OR ("mean platelet volume"):ti,ab,kw OR (platelet activation):ti,ab,kw |

**Appendix 3**

Excluded articles (N=23) after the second phase of the search and selection procedure, based on the full text reading of the articles.

| **Reason of exclusion** | **Articles** |
| --- | --- |
| **Lack of periodontitis case definition based on review eligibility criteria** | 1. Androsz-Kowalska et al. (1) 2. Antonopoulou et al. (2) 3. Fredman et al. (3) 4. Hu et al. (4) 5. Kumar et al. (5) 6. Moradi Haghgoo et al. (6) 7. Prabhahar et al. (7) 8. Rai et al. (8) 9. Romandini et al. (9) 10. Torrungruang et al. (10) 11. Zhou et al. (11) |
| **Participants < 18 years old** | 1. Dosumu et al. (12) 2. Iqbal et al. (13) 3. López et al. (14) 4. Lu et al. (15) 5. Shi et al. (16) |
| **Lack of outcome of interest** | 1. Antonopoulou et al. (2) 2. Krause et al. (17) 3. Malik et al. (18) 4. Zhang et al. (19) 5. Zhou et al. (20) |
| **Lack of non-periodontitis group** | 1. Hu et al. (4) 2. Lamster et al. (21) 3. Zhou et al. (20) |
| **Lack of exposure of interest** | 1. Chen et al. (22) 2. Lamster et al. (20) |
| **Lack of data for periodontitis case definition criteria** | 1. Krause et al. (23) |

**References Appendix 3:**

1. Androsz-Kowalska O, Jankowski K, Rymarczyk Z, Kowalski J, Pruszczyk P, Górska R. Correlation between clinical parameters of periodontal disease and mean platelet volume in patients with coronary artery disease: a pilot study. Kardiol Pol. 2013;71(6):600–605.

2. Antonopoulou S, Tsoupras A, Baltas G, Kotsifaki H, Mantzavinos Z, Demopoulos CA. Hydroxyl-platelet-activating factor exists in blood of healthy volunteers and periodontal patients. Mediators Inflamm. 2003;12(4):221–227.

3. Fredman G, Oh SF, Ayilavarapu S, Hasturk H, Serhan CN, Van Dyke TE. Impaired Phagocytosis in Localized Aggressive Periodontitis: Rescue by Resolvin E1. PLoS One. 2011;6(9):e24422.

4. Hu M, Zhang W, Shi Z, et al. Data on hematological parameters and generalized severe periodontitis in the United States. Data Brief. 2024;52:110010.

5. Kumar BP, Khaitan T, Ramaswamy P, Sreenivasulu P, Uday G, Velugubantla RG. Association of chronic periodontitis with white blood cell and platelet count - A Case Control Study. J Clin Exp Dent. 2014;6(3):e214-217.

6. Moradi Haghgoo J, Torkzaban P, Farhadian M, Rabienejad N, Moosavi Sedeh SA. Hematologic tests and their association with the severity of COVID-19 and periodontitis in hospitalized patients: a case–control study. BMC Oral Health. 2023;23(1):473.

7. Prabhahar CS, Niazi KT, Prakash R, Yuvaraj A, Goud S, Ravishekar P. Estimation of salivary β-glucuronidase activity as a marker of periodontal disease: A case control study. J Int Soc Prev Community Dent. 2014;4(Suppl 3):S193–198.

8. Rai B, Kaur J, Anand SC. Possible relationship between periodontitis and dementia in a North Indian old age population: a pilot study. Gerodontology. 2012;29(2):e200–205.

9. Romandini M, Laforí A, Romandini P, Baima G, Cordaro M. Periodontitis and platelet count: A new potential link with cardiovascular and other systemic inflammatory diseases. J Clin Periodontol. 2018;45(11):1299–1310.

10. Torrungruang K, Ongphiphadhanakul B, Jitpakdeebordin S, Sarujikumjornwatana S. Mediation analysis of systemic inflammation on the association between periodontitis and glycaemic status. J Clin Periodontol. 2018;45(5):548–556.

11. Zhou C, Liu Y, Bai J, Luo Y, Song J, Feng P. Mean platelet volume is associated with periodontitis: a cross-sectional study. BMC Oral Health. 2024;24(1):461.

12. Dosumu EB, Arowojolu MO, Akande OO, Akingbola TS. Hematological values in juvienile periodontitis patients in  Ibadan, Nigeria. Afr J Biomed Res. 2002;5:141–143.

13. Iqbal PS, Khan SN, Haris M, Narayanan M, Laju S, Kumar SS. Assessment of Systemic Inflammatory Markers in Patients with Aggressive Periodontitis. J Int Oral Health. 2015;7(Suppl 2):48–51.

14. López R, Loos BG, Baelum V. Hematological features in adolescents with periodontitis. Clin Oral Investig. 2012;16(4):1209–1216.

15. Lu R, Li W, Wang X, Shi D, Meng H. Elevated neutrophil-to-lymphocyte ratio but not platelet-to-lymphocyte ratio is associated with generalized aggressive periodontitis in a Chinese population. J Periodontol. 2021;92(4):507–513.

16. Shi D, Meng H, Xu L, et al. Systemic Inflammation Markers in Patients With Aggressive Periodontitis: A Pilot Study. J Periodontol. 2008;79(12):2340–2346.

17. Krause S, Brachmann P, Brandes C, Lösche W, Hoffmann T, Gängler P. Aggregation behaviour of blood granulocytes in patients with periodontal disease. Arch Oral Biol. 1990;35(1):75–77.

18. Malik N, Kalburgi V, Malik S, Srivastava S, Verma H, Rai KS. Effect of Non-surgical Periodontal Therapy on Platelet-to-Lymphocyte Ratio and Neutrophil-to-Lymphocyte Ratio in Chronic Periodontitis. J Young Pharm. 2022;14(2):227–230.

19. Zhang J, Li W, Lu H, Lu R, Zhan Y, Meng H. Interactions of periodontal pathogens with platelets in the gingival crevicular fluid of patients with periodontitis. J Clin Periodontol. 2022;49(9):922–931.

20. Zhou H, Zhang S, Miao D, Cao R. U-shaped association between pan-immune-inflammation value and periodontitis: NHANES 2009-2014. J Periodontol. 2024; Epub ahead of print.

21. Lamster IB, Kaufman E, Grbic JT, Winston LJ, Singer RE. Beta-glucuronidase activity in saliva: relationship to clinical periodontal parameters. J Periodontol. 2003;74(3):353–359.

22. Chen WA, Fletcher HM, Payne KJ, et al. Platelet and neutrophil responses to Porphyromonas gingivalis in human whole blood. Mol Oral Microbiol. 2021;36(3):202–213.

23. Krause S, Brachmann P, Lösche W, Hoffmann T, Gängler P. Defective function of blood granulocytes in patients with diabetes mellitus-associated marginal periodontitis. Folia Biol (Praha). 1992;38(6):358–363.

**Appendix 4**

Overview of the studies included for the data extraction and analysis.

| Study | Location;  Study design | Sample size;  Sex (male/female);  Age of participants | Periodontitis case definition | Variable of interest |
| --- | --- | --- | --- | --- |
| *I. Acharya et al. 2019 (57)* | India  Cross-sectional | Control: n = 30  15M/15F  Periodontitis: n = 30  15M/15F  Age range: 18-55 years | 1) presence $\geq$20 natural teeth,  2) > 30% of the examined sites with PPD ≥ 4 mm and CAL ≥ 2 mm; positive for bleeding on probing; radiographic evidence of bone loss | PC |
| *II. Al-Rasheed 2012 (58)* | Saudi Arabia  Cross-sectional | Control: n = 50  29M/21F  34.00 ± 6.2 years  Periodontitis: n = 50  32M/18F  34.35 ± 7.82 years | > 30% of teeth with PPD ≥ 4 mm and CAL ≥ 2 mm. | PC |
| *III. Anand et al. 2016 (50)* | India  Case-control | Control: n = 63  36M/27F  30.52 ± 7.59 years  GAgP: n = 75  38M/37F  33.47 ± 8.41 years | 1) PPD and CAL of ≥ 5 mm on ≥ 8 permanent teeth of which ≥ 3 teeth were not permanent first molars or incisors,  2) were systemically healthy,  3) showed familial aggregation (presence or history of periodontal disease in at least one other family  member) | PC |
| *IV. Assinger et al. 2011, 2012 (4,44); Laky et al. 2011 (45)* | Austria  Cross-sectional | Control: n = 19  10M/9F  40 ± 2.49 years  Periodontitis: n = 26  13M/13F  42 ± 1.68 years | ≥ 6 teeth with PPD ≥ 6 mm and CAL ≥ 5 mm | Platelet activation based on:  i. plasma levels and surface expression of P-selectin,  ii. surface expression and plasma levels of CD40L,  iii. GPIIb/IIIa activation (% of + cells),  iv. phosphatidylserine (% of + cells),  v. intracellular VASP-phosphorylation (% of + cells) |
| *V. Bhattacharya et al. 2022 (56)* | India  Cross-sectional | Control: n = 40  19M/21F  36.93 ± 8.24 years  Periodontitis: n =40  21M/19F  41.8 ± 10.28 years | ≥ 1 site with PPD ≥ 6 mm and radiographic evidence of bone loss on an orthopantomogram | 1) PC  2) MPV |
| *VI. Brousseau-Nault et al. 2017 (22)* | Canada  Cross-sectional | Control: n = 7  1M/6F  43.4 ± 7.8 years  Periodontitis: n = 4  3M/1F  51.2 ± 13.5 years | Radiographic evidence of alveolar bone loss and PPD ≥ 5 mm at ≥ 30% of sites | Platelet activation based on PF4 concentration in plasma and total platelet PF4 content |
| *VII. Dolma et al. 2020 (63)* | India  Cross-sectional | Control non-smokers: n = 25  sex not available  33.76 ± 6.32 years  Control smokers: n = 25  sex not available  30.72 ± 5.03  Periodontitis non-smokers: n = 25  sex not available  41.96 ± 6.36  Periodontitis smokers: n = 25  sex not available  40.70 ± 5.35 | ≥ 1 site with PPD > 5 mm and CAL ≥ 4 mm | MPV |
| *VIII. Lee et al. 2023 (59)* | Republic of Korea  Cross-sectional | Control: n = 11  7M/4F  53.84 ± 24.26 years  Periodontitis: n = 10  7M/3F  66.47 ± 8.34 years  Periodontitis & DM type 2: n = 6  4M/2F  54.39 ± 8.09 years | Classification criteria based on 2017 World Workshop Classification System  for periodontal and peri-implant diseases and conditions | Platelet activation based on platelets transcriptional profiles |
| *IX. Marcaccini et al. 2009 (51)* | Brazil  Cross-sectional | Control: n = 20  6M/14F  42.8± 5.1 years  Periodontitis: n = 25  9M/16F  44.9± 5.5 years | ≥ 2 teeth with PPD ≥ 5 mm, CAL ≥ 6 mm, and radiographic evidence of alveolar bone loss | Platelet activation based on:  1) CD40 ligand  2) sP-selectin |
| *X. Mishra et al. 2023 (48)* | India  Cross-sectional | Control: n = 315  158M/157F  34 (30, 37) years  Periodontitis: n = 315  166M/149F  34 (30, 48) years | Classification criteria based on 2017 World Workshop Classification System  for periodontal and peri-implant diseases and conditions | PC |
| *XI. Mishra et al. 2022 (53)* | India  Case-control | Control: n = 40  21M/19F  31.50 ± 5.31 years  Periodontitis: n = 108  59M/49F  30.67 ± 4.89 years | Classification criteria based on 2017 World Workshop Classification System  for periodontal and peri-implant diseases and conditions | PC |
| *XII. Mutthineni et al. 2021 (61)* | India  Cross-sectional | Control: n = 25  sex not available  Moderate Periodontitis: n = 25  sex not available  Severe Periodontitis: n = 25  sex not available  Age range: 35-50 years | Moderate periodontitis: ≥ 2 interproximal sites with CAL ≥ 4 mm (not on the same tooth) or ≥ 2 interproximal sites with PPD ≥ 5 mm (not on the same tooth)  Severe periodontitis: ≥ 2 interproximal sites with CAL ≥ 6 mm and ≥ 1 interproximal site with PPD ≥ 5 mm | 1) MPV  2) Platelet distribution width  3) Plateletcrit |
| *XIII. Nibali et al. 2019I (47)* | United Kingdom  Cross-sectional | Control: n = 225  104M/121F  37.65 ± 11.52 years  AgP: n = 125  44M/81F  33.79 ± 6.18 years  CP: n = 121  50M/71F  45.12 ± 10.04 years | 1) ≥ 1 site with ≥ 5 mm PPD and CAL (excluding third molars and distal surfaces of second molars)  2) AgP:  systemically healthy patients, aged ≤ 45 years, with ≥ 3 teeth with CAL ≥ 6 mm and BOP, and with familial aggregation | 1) MPV  2) PC |
| *XIV. Nibali et al. 2019II (47)* | United Kingdom  Cross-sectional | Control: n = 206  89M/117F  38.25 ± 11.51 years  AgP: n = 127  48M/79F  29.72 ± 7.59 years | 1) systemically healthy patients,  2) aged < 35 years or with radiographic evidence of rapid bone destruction,  3) ≥ 2 permanent teeth of which ≥ 1 first molar or incisor with interproximal CAL and PPD ≥ 5 mm and radiographic bone loss ≥ 30% of root length,  4) familial aggregation | 1) MPV  2) PC |
| *XV. Nicu et al. 2009 (21)* | The Netherlands  Cross-sectional | Control: n = 18  6M/12F,  40.8 (36.1-45.4) years  Periodontitis n = 19  7M/12F  42.0 (37.5-46.4) years | 1) Generalized gingival inflammation and deepened periodontal pockets,  2) ≥ 8 teeth with ≥ 30% radiographic bone loss of the root length | 1) PC  2) Platelet activation as expressed on plasma levels of sP-selectin and sCD40L  3) Platelet activation as expressed with mean fluorescence intensity (MFI) of P-selectin, PAC-1, CD63, binding of PAC-1 ability  4) Platelet activation based on the formation of platelets-neutrophils and platelets-monocytes complexes |
| *XVI. Papapanagiotou et al. 2009 (20)* | The Netherlands  Cross-sectional | Control: n = 35  12M/23F  37.6 ± 9.1 years  Periodontitis: n = 85  41M/44F  44.7 ± 8.8 years | 1) Generalized gingival inflammation and deepened periodontal pockets,  2) Radiographic distance > 3 mm between the CEJ and the alveolar bone crest | Platelet activation as based on plasma levels of sP-selectin, sCD40 Ligand |
| *XVII. Perumal et al. 2014 (60)* | India  Cross-sectional | Control: n = 40  sex not available  Generalized Chronic Periodontitis: n = 40  sex not available  Age range: 35-60 years | 1) Bleeding on probing  2) CAL ≥ 5 mm  3) ≥ 20 teeth present  4) PPD in mm  5) Poor oral hygiene | 1) PC  2) Platelet form  3) Platelets aggregation  4) Serum P-selectin levels |
| *XVIII. Siqueira et al. 2013 (62)* | Brazil  Cross-sectional | Control: n = 8  4M/4F  44.5 ± 1.2 years  Periodontitis: n = 8  3M/5F  49.5 ± 2.1 years | ≥ 3 teeth with CAL ≥ 4 mm and PPD ≥ 5 mm | Platelet activation based on:  1) L-arginine influx  2) Basal NOS activity  3) Arginase activity  4) Platelet expression of eNOS, iNOS, arginase II, α_1_, β_1_ GCs and PDE 5  5) cGMP  6) Platelet aggregation  7) Platelet ROS  8) SOD activity  9) Catalase activity |
| *XIX. Temelli et al. 2018 (49)* | Turkey  Cross-sectional | Control: n = 36  10M/16F  age range 33-65 years  Periodontitis: n = 41  27M/14F  age range 42-71 years | Classification criteria according to the  1999 International Workshop for the Classification of Periodontal Disease and Conditions | 1) MPV  2) PC |
| *XX. Ustaoglu et al. 2020 (55)* | Turkey  Cross-sectional | Control: n = 57  26M/31F  35.6±7.0 years  Stage III Periodontitis: n = 57  29M/28F  37.4±7.0 years  Age range: 18-50 years | 1) PPD ≥ 6 mm and interdental CAL ≥ 5 mm  2) tooth loss of ≤ 4 teeth due to periodontitis  3) radiographically detected bone loss reaching the mid-third of the root and beyond | 1) MPV  2) Plateletcrit  3) PC |
| *XXI. Wang et al. 2015 (52)* | China  Cross-sectional | Control: n = 45  25M/20F  34.8 ± 12.0 years  Periodontitis: n = 45  24M/21F  35.3 ± 10.3 years | 1) ≥ 2 interproximal sites with CAL ≥ 6 mm, not on the same tooth  2) ≥ 1 interproximal site with PPD ≥ 5 mm | 1) MPV  2) PC  3) Platelet large cell ratio |
| *XXII. Wang et al. 2019 (46); Zhan et al. 2016, 2017 (23,24)* | China  Case-control | Control: n = 139  53M/86F  27.15 ± 4.26 years  GAgP: n = 224  85M/139F  27.33 ± 4.60 years | 1) onset of periodontal disease at < 35 years  2) ≥ 20 functional teeth remaining in the mouth  3) ≥ 8 teeth with PPD > 5 mm and CAL > 3 mm, ≥ 3 of which were not first molars or incisors  4) radiographic alveolar bone loss | 1) PC  2) MPV  3) Platelet distribution width  4) Platelet large cell ratio |
| *XXIII. Zhao et al. 2024 (54)* | United States of America  Cross-sectional | Control: n = 5091  2240M/2851F  48 (39.00, 60.00)  Periodontitis: n = 4379  2611M/1768F  55 (45.00, 65.00) | 1) ≥ 2 adjacent sites with CAL ≥ 3 mm  2) ≥ 2 adjacent sites with PPD ≥ 4 mm (not on the same tooth) or ≥ 1 site with PPD ≥ 5 mm | PC |

AgP: Aggressive Periodontitis, CAL: Clinical Attachment Levels, CD40L: Cluster of Differentiation 40 Ligand, CD63: Cluster of Differentiation 63 antigen, CEJ: Cementoenamel Junction, cGMP: cyclic Guanosine Monophosphate , CP: Chronic Periodontitis, DM: Diabetes Mellitus, eNOS: endothelial Nitric Oxide Synthase, F: female, GAgP: Generalized Aggressive Periodontitis, GCs: Guanylate Cyclase subunits, GPIIb/IIIa: Glycoprotein IIb/IIIa (also known as integrin αIIbβ3), iNOS: inducible Nitric Oxide Synthase, M: male, MFI: Mean Fluorescence Intensity, MPV: Mean Platelet Volume, NOS: Nitric Oxide Synthase, PAC-1: first Procaspase Activating Compound, PC: Platelet count, PDE 5: Phosphodiesterase 5, PF4: Platelet Factor 4, PPD: Probing Pocket Depth, ROS: Reactive Oxygen Species, sCD40L: soluble Cluster of Differentiation 40 Ligand, SOD: Superoxide Dismutase, sP-selectin: soluble P-selectin, VASP: Vasodilator-Stimulated Phosphoprotein, WBC: White Blood Cell.

**Appendix 5**

Assessment of clinical and methodological heterogeneity

The participant counts varied considerably, with the smallest study conducted by Brousseau-Nault et al. (22) including just 11 individuals, while the largest, performed by Zhao et al. (54), involved 9470 participants.

Geographically, eight studies were carried out in south Asia, all of them in India, (48,50,53,56,57,60,61,63), five studies were carried out in Europe (4,20,21,44,45,47), three in east Asia (23,24,46,52,59) and in west Asia (49,55,58), two in south America (51,62) and in north America (22,54).

Regarding the age range of participants, most investigations providing only mean ages (4,22,44,45,50,51,56,58,59,63) or medians (48,54), making it impossible to ascertain an exact age range for the study populations.

Sex information was not specified in three studies (60,61,63), while the remaining reported varied ratios of male and female participants.

Apart from three investigations (49,54,59), all participants were in good systemic health. Lee et al. (59) included individuals with type 2 diabetes mellitus, Zhao et al. (54) patients with diabetes mellitus and/or hypertension, and Temelli et al. (49) studied patients with coronary artery disease.

Seven studies comprised smokers (20,21,47,49,54,63), while the smoking status of the participants in two studies remained unclear (59,62). The remaining studies excluded smokers (4,22,44,45,50,56-58).

Eleven studies employed PPD and CAL measurements for defining periodontitis (4,44,45,47,49,50,52,54,58,60-63). Eight studies additionally relied on radiographic evidence of bone loss (48,51,53,57,59), and four used PPD and radiographic bone loss (20-22,56).

Six studies adopted the criteria from the 1999 Consensus Classification (64) for periodontitis diagnosis (23,24,46,47,49,50,60). Among these, three diagnosed patients with aggressive periodontitis (23,24,46,47,50), two with chronic periodontitis (49,60), and one study considered both forms (47). Four investigations (48,53,55,59) used the criteria of the 2017 Consensus Classification (65,93).

For periodontitis case definition the threshold for PPD ranged from ≥ 4 mm (54,57,58) to ≥ 6 mm (4,23,24,44-46,55,56) and for CAL from ≥ 2 mm (57-58) to ≥ 6 mm (51,52). Further, three studies defined periodontitis with a threshold of ≥ 30% radiographic bone loss (21,47,55), while Papapanagiotou et al. (20) used > 3 mm radiographic bone loss.

Four studies defined periodontitis with the presence of ≥ 1 affected site (47,52,56,63), two required ≥ 2 affected sites (54,61), and three specified > 30% affected sites (22,57,58). One study defined periodontitis with the presence of ≥ 1 affected tooth (51), 3 studies required ≥ 3 affected teeth (47,62), one demanded ≥ 6 teeth (4,44,45), one specified ≥ 8 affected teeth (20,21), 2 studies ≥ 8 affected teeth of which ≥ 3 teeth were not first molars or incisors (23,24,46,50) and one ≥ 2 teeth of which ≥ 1 tooth first molar or incisor (47). Two investigations did not provide specific information regarding the number of sites or teeth used as thresholds for periodontitis diagnosis (20,60).

In eight of the studies a full-mouth periodontal examination was conducted (48,47,49,51,55,57,58). In eight studies the periodontal examination excluded the third molars (21,23,24,46,50,52-54,62,63) and six did not provide information on the number of teeth examined (4,22,44,45,56,59-61).

Three studies used a case-control design (23,24,46,50,53), while the remaining a cross-sectional (4,20-22,44,45,47-49,51,52,54-63).

Fifteen studies assessed PC (21,23,24,46-50,52-58,60), while nine the MPV (23,24,46,47,49,52,55,56,61,63). Five studies investigated the expression of P-selectin (4,20,21,44,45,51,60), and four explored the expression of cluster of differentiation 40 ligand (4,20,21,44,45,51).

Two studies evaluated the platelet large cell ratio (23,24,46,52) and another two the plateletcrit (55,61). Nicu et al. (21) examined the formation of platelets-monocytes and platelets-neutrophils complexes, whilst two other studies the platelets aggregation (60,62).

Mutthineni et al. (61) and Wang et al. (52) studied the platelets distribution width, while Perumal et al. (60) the platelets form. Assinger et al. (4,44) explored glycoprotein IIb/IIIa activation, phosphatidylserine expression, and intracellular vasodilator-stimulated phosphoprotein phosphorylation. Additionally, Lee et al. (59) analyzed the transcriptional profiles of platelets, while Broussseau-Nault et al. (22) the platelet factor 4 (PF4) concentration in plasma and PF4 total platelet content.

Nicu et al. (21) assessed platelets activation based on the mean fluorescence intensity of first procaspase activating compound (PAC-1), cluster of differentiation 63 antigen and PAC-1 binding ability. Meanwhile, Siqueira et al. (62) measured activation through L-arginine influx, basal, endothelial, and inducible nitric oxide synthase, catalase, superoxide dismutase and arginase activity, as well as the expression of cyclic guanosine monophosphate, arginase II, α_1_, β_1_ guanylate cyclase subunits and phosphodiesterase 5, along with platelets concentrations of reactive oxygen species.

**Appendix 6**

A. Modified Newcatle – Ottawa quality assessment of the cross-sectional studies

| **Study** | **Selection^a^**  **(max 5)** | **Comparability^b^**  **(max 2)** | **Outcome**  **(max 3)** | **Score** | **Judgement** |
| --- | --- | --- | --- | --- | --- |
| Acharya et al. (57) | ★★★ |  | ★★★ | 6/10 | M |
| Al-Rasheed (58) | ★★★ |  | ★★★ | 6/10 | M |
| Assinger et al. (4,44); Laky et al. (45) | ★★★ | ★★ | ★★★ | 8/10 | L |
| Bhattacharya et al. (56) | ★★★ |  | ★★★ | 6/10 | M |
| Brousseau-Nault et al. (22) | **★★★** |  | ★★★ | 6/10 | M |
| Dolma et al. (63) | **★★★** |  | ★★★ | 6/10 | M |
| Lee et al. (59) | **★★★** |  | **★★★** | 6/10 | M |
| Marcaccini et al. (51) | ★★★★ |  | ★★★ | 7/10 | L |
| Mishra et al. (48) | ★★★★ |  | ★★★ | 7/10 | L |
| Mutthineni et al. (61) | ★★ |  | ★★★ | 5/10 | M |
| Nibali et al. I (47) | ★★★★ | **★★** | ★★★ | 9/10 | L |
| Nibali et al. II (47) | ★★★★ | **★★** | ★★★ | 9/10 | L |
| Nicu et al. (21) | ★★★ | **★★** | ★★★ | 8/10 | L |
| Papapanagiotou et al. (20) | ★★★ | **★** | ★★★ | 7/10 | L |
| Perumal et al. (60) | ★★★ |  | ★★★ | 6/10 | M |
| Siqueira et al. (62) | ★★★ |  | ★★★ | 6/10 | M |
| Temelli et al. (49) | ★★★★ |  | ★★★ | 7/10 | L |
| Ustaoglu et al. (55) | ★★★★ |  | ★★★ | 7/10 | L |
| Wang et al. (52) | ★★★ | ★★ | ★★★ | 8/10 | L |
| Zhao et al. (54) | ★★★★ | **★★** | ★★★ | 9/10 | L |

L = Low, M = Medium, H = High

Studies with at least seven stars were categorized as having a low risk of bias, those with five or six stars as moderate risk of bias and studies with fewer than five stars as high risk of bias.

a: 2 stars were awarded, if the study provided a periodontitis case definition, based on clinical examination evaluating probing pocket depth in combination with clinical attachment loss and/or radiographic bone loss.

b: 2 stars were awarded, if the individuals in case and control groups were matched or if the analysis was adjusted for age (1 star) and sex (1 star).

B. Newcastle – Ottawa quality assessment of the case-control studies

| **Study** | **Selection^a^**  **(max 4)** | **Comparability^b^**  **(max 2)** | **Outcome**  **(max 3)** | **Score** | **Judgement** |
| --- | --- | --- | --- | --- | --- |
| Anand et al. (50) | ★★★ | ★★ | ★★★ | 8/9 | L |
| Mishra et al. (53) | ★★★★ |  | ★★★ | 7/9 | L |
| Wang et al. (46); Zhan et al. (23,24) | ★★★ | ★★ | ★★★ | 8/9 | L |

For interpretation of the judgement see appendix 6A

a: 1 star was awarded, if the study provided a periodontitis case definition, based on clinical examination evaluating probing pocket depth in combination with clinical attachment loss and/or radiographic bone loss.

b: 2 stars were awarded, if the individuals in case and control groups were matched or if the analysis was adjusted for age (1 star) and sex (1 star).

**Appendix 7**

Overview of the outcomes and original authors conclusions of the included studies

| Study | Outcomes | Conclusions |
| --- | --- | --- |
| *I. Acharya et al. (57)* | PC:  Periodontitis: 320.33 ± 108.11 x10^9^/L  Control: 281.93 ± 99.04 x10^9^/L | The PC was elevated in periodontitis group versus the control group. |
| *II. Al-Rasheed (58)* | PC:  Periodontitis: 290.73 ± 56.56 x10^9^/L  Control: 223.37 ± 50.27 x10^9^/L  p < 0.001 | Periodontitis may elevate PC compared to healthy participants which might be considered one of the  mechanisms that explain how periodontitis is linked to the development of atherosclerosis and cardiovascular disease. |
| *III. Anand et al. (50)* | PC:  GAgP: 229.81 ± 69.60 x10^9^/L  Control: 238.38 ± 62.24 x10^9^/L  p = 0.447 | The PC was slightly higher in the control group than in the GAgP group, but the differences were not statistically significant. |
| *IV. Assinger et al. (4,44); Laky et al. (45)* | 1) Plasma levels of soluble P-selectin:  Periodontitis: 93.29 ng/ml [51.76-110.72]  Control: 48.51 ng/ml [42.35-77.37]  p = 0.019  2) No significant difference in surface expression of P-selectin and CD40L on platelets between periodontitis and control  3) Plasma levels of soluble CD40L:  Periodontitis: 1.65 ng/ml [0.70-4.79]  Control: 1.38 ng/ml [0.52-19.3]  no significant difference  4) No significant difference in GPIIb/IIIa activation and phosphatidylserine levels between periodontitis and control  5) Significant difference in intracellular VASP-phosphorylation between periodontitis and control | The data indicates that periodontitis and periodontitis-associated pathogens are associated with increased plasma levels of P-selectin and CD40L and interfere with VASP-phosphorylation in human platelets. As phosphorylated VASP represents a negative regulator of platelet function, this may contribute to platelet activation that can be observed in patients with periodontitis. |
| *V. Bhattacharya et al. (56)* | 1) PC:  Periodontitis^a^: 291 ± 95 x10^9^/L  Control^a^: 203 ± 0.66 x10^9^/L  p < 0.001  2) MPV:  Periodontitis: 12.7 ± 1.41 fL  Control: 12.74 ± 1.2 fL  p = 0.898 | Increased levels of PC depict the inflammatory state and destructive nature of periodontitis. |
| *VI. Brousseau-Nault et al. (22)* | 1) The circulating concentration of PF4 was ~2 times higher (p < 0.05) in patients with generalized severe periodontitis, compared to non-periodontitis individuals  2) Resting platelets isolated from the whole blood of patients with severe periodontitis contained more PF4 than platelets from the other two patient groups although this was not statistically significant p > 0.05 | Periodontitis is associated with increased platelet activation and PF4 release, both locally and systemically. |
| *VII. Dolma et al. (63)* | MPV:  Periodontitis smokers = 10.06 ± 0.53 fL  Periodontitis non-smokers = 9.89 ± 0.53 fL  Control smokers = 11.12 ± 0.72 fL  Control non-smokers = 10.44 ± 0.35 fL  Statistically significant difference between all the groups (p < 0.05) except between the two periodontitis groups (p = 0.269). | MPV was decreased among smokers with chronic periodontitis. |
| *VIII. Lee et al. (59)* | 1) Patients with periodontitis, and patients with periodontitis and DM type 2 showed higher level of activation and coagulation signals versus controls.  2) Platelets from patients with periodontitis showed higher expression levels of genes for Rho GTPase effectors compared to controls.  3) Platelets from patients with periodontitis and DM type 2 showed higher expression level of genes related in oxidative phosphorylation and cellular responses to stress than the controls.  4) Platelets from patients with periodontitis showed higher levels of genes for hemostasis and platelet receptors compared to patients with periodontitis and DM type 2. | The results suggest that periodontitis contributes to the establishment of blood coagulation through platelet dysregulation. |
| *IX. Marcaccini et al. (51)* | 1) CD40 ligand:  Periodontitis: 2.0 ± 0.9 ng/ml  Control: 1.4 ± 0.3 ng/ml  p = 0.0098  2) sP-selectin:  Periodontitis: 91.8 ± 79.2 ng/ml  Control: 66.8 ± 46.2 ng/ml  No significant difference | The results suggest that the CD40 ligand may also be increased in patients with periodontitis. The CD40 ligand might indicate a more subtle change in the platelets that takes place before changes in fibrinogen can be observed. |
| *X. Mishra et al. (48)* | PC:  Periodontitis: 278.37 ± 78.55 x10^9^/L  Control: 268.55 ± 66.25 x10^9^/L  p = 0.09 | Although higher in periodontitis group, PC did not attain statistical significance. |
| *XI. Mishra et al. (53)* | PC:  Periodontitis: 298.69 ± 84.17 x10^9^/L  Control: 286.28 ± 82.69 x10^9^/L  p = 0.4248 | The present study reported no prominent differences in PC between patients with periodontitis and periodontally healthy individuals. |
| *XII. Mutthineni et al. (61)* | 1) MPV:  Moderate Periodontitis: 11.44 ± 1.40 fL  Severe Periodontitis: 12.17 ± 0.53 fL  Control: 9.13 ± 0.93 fL  P < 0.001, severe > moderate > control  2) PDW:  Moderate Periodontitis: 10.61 ± 1.58  Severe Periodontitis: 10.87 ± 1.40  Control: 10.51 ± 2.71  p < 0.001, significant difference between patients with periodontitis and control.  3) PCT:  Moderate Periodontitis: 0.30 ± 0.06  Severe Periodontitis: 0.42 ± 0.09  Control: 0.19 ± 0.04  p < 0.001, severe > moderate > control | Platelet indices MPV, PCT, and PDW can be used as simple, practical, and cost‑effective biomarker for periodontitis. The chronic inflammatory response triggered by periodontitis results in platelet aggregation and platelet activation which leads to a change in platelet number, size, and shape. This leads to a change in the platelet indices, thus proving as a reliable biomarker for the assessment of both periodontal and cardiovascular disease. |
| *XIII. Nibali et al. I (47)* | 1) MPV:  Males: AgP: 11.13 ± 0.90 fL  CP: 11.22 ± 1.05 fL  Control: 10.91 ± 0.87 fL  p = 0.053 (AgP+CP versus Control)  Females: AgP: 10.85 ± 0.84 fL  CP: 11.13 ± 0.94 fL  Control: 10.85 ± 0.89 fL  p = 0.278 (AgP+CP versus Control)  2) PC:  Males: AgP^a^: 230.32 ± 56.33 x10^9^/L  CP^a^: 227.04 ± 52.07 x10^9^/L  Control^a^: 233.80 ± 43.98 x10^9^/L  p = 0.467 (AgP+CP versus Control)  Females: AgP^a^: 263.40 ± 56.59 x10^9^/L  CP^a^: 256.80 ± 60.99 x10^9^/L  Control^a^: 262.84 ± 48.86 x10^9^/L  p = 0.717 (AgP+CP versus Control) | The proposed association between presence of periodontitis and increased number of platelets could not be confirmed in this study, although a slight increase was found in AgP patients compared with controls. |
| *XIV. Nibali et al. II (47)* | 1) MPV:  Males: AgP: 10.49 ± 1.08 fL  Control: 11.03 ± 0.89 fL  p = 0.003  Females: AgP: 10.57 ± 1.15 fL  Control: 10.87 ± 0.81 fL  p = 0.042  2) PC:  Males: AgP^a^: 258.77 ± 54.52 x10^9^/L  Control^a^: 235.75 ± 40.70 x10^9^/L  p = 0.006  Females: AgP^a^: 267.73 ± 68.44 x10^9^/L  Control^a^: 281.01 ± 61.13 x10^9^/L  p = 0.157 | The proposed association between presence of periodontitis and increased number of platelets could not be confirmed in this study, although a slight increase was found in AgP patients compared with controls. |
| *XV. Nicu et al. (21)* | 1) PC:  Periodontitis: 276.6 x10^9^/L (253.4–299.8)  Control: 240.3 x10^9^/L (216.5-264.2)  p = 0.040  2) sP-selectin:  Periodontitis: 56.9 ± 17.5 ng/mL  Control: 49.5 ± 13.6 ng/mL  p = 0.182  3) sCD40L:  Periodontitis: 217 ± 134 pg/mL  Control: 155 ± 114 pg/mL  p = 0.159  4) no significant differences between patients with periodontitis and controls in the MFI of activation markers at their unstimulated blood samples, except binding of PAC-1 ability that was significantly higher in patients with periodontitis  5) no significant differences in platelets-monocytes and platelets-neutrophil complexes formation between patients with periodontitis and control | The findings may in part explain the increased relative risk for cardiovascular events in patients with periodontitis. |
| *XVI. Papapanagiotou et al. (20)* | 1) sP-selectin:  Periodontitis: 82.9 ± 24.7 ng/mL  Control: 58.1 ± 26.1 ng/mL  p = 0.0002  2) sCD40L:  Periodontitis: 794 ± 647 pg/mL  Control: 624 ± 564 pg/mL  p = 0.180 | Periodontitis is associated with increased platelet activation. Since platelet activation contributes to a pro-coagulant state and constitutes a risk for atherothrombosis, platelet activation in periodontitis may partly explain the epidemiological association between periodontitis and CVDs. |
| *XVII. Perumal et al. (60)* | 1) PC:  Periodontitis^a^: 281.25 ± 84.5 x10^9^/L  Control^a^: 200.98 ± 56.9 x10^9^/L  p < 0.001  2) Increased P-selectin expressions was positively associated with CAL, p <0.001  3) Platelet form:  Small form: Periodontitis: 39.08 ± 21.59  Control: 75.83 ± 14.24%  p < 0.001  Big form: Periodontitis: 0.48 ± 1.3%  Control: 0.80 ± 0.35%  p = 0.075  Spider form: Periodontitis: 59.32 ± 23.42%  Control: 23.88 ± 14.13%  p < 0.001  4) Platelets aggregation:  Grade I: Periodontitis: 22.2%  Control: 77.8%  Grade II: Periodontitis: 24.3%  Control: 75.5%  Grade III: Periodontitis: 84.4%  Control: 15.6%  Grade IV: Periodontitis: 100%  Control: 0%  5) Within the group correlation:  Periodontitis: Grade III (67.5%), Grade II (22.3%), Grade and Grade IV (5.0% each)  Control: Grade II (70%), Grade I (17.5%), Grade III (12.5%) and Grade IV (0%)  p < 0.001 | The results of the study showed higher number of spider forms and significant pathological aggregation pattern in patients with periodontitis, which indicates activation of platelets.  An elevated serum P-selectin level was seen in patients with periodontitis compared to the control.  Also, P-selectin expression increased in severe periodontitis than moderate periodontitis.  These products have the potential to activate platelets which in turn can release an arsenal of potent inflammatory and mitogenic substances leading to an altered endothelial function (chemotaxis and adhesion). |
| *XVIII. Siqueira et al. (62)* | 1) Total L-arginine transport and via the y+L system were increased in patients with periodontitis compared to the controls (p < 0.05)  2) Basal NOS activity (pmol/10^8^ cells/min,):  Periodontitis: 0.11 ± 0.02  Control: 0.12 ± 0.02  No significant difference  3) Arginase activity:  Periodontitis: 21.7 [4.2– 49.9] pmol urea/mg protein/2 h  Control: 40.1 [6.1–464.0] pmol urea/mg protein/2 h  No significant difference  4) Platelet expression:  **eNOS** controls: 0.9 ± 0.4 vs. periodontitis: 1.9 ± 0.6 arbitrary units, no significant difference  **iNOS** controls: 0.6 ± 0.2 vs. periodontitis: 1.6 ± 0,6 arbitrary units, no significant difference  **arginase II** controls: 30.3 ± 7.3 vs. periodontitis: 54.3 ± 15.7 arbitrary units, no significant difference  5) Basal cGMP levels in platelets from patients with periodontitis were significantly decreased compared to controls  6) Platelet aggregation assays (%) induced by fibrillar collagen (4μg/mL):  Periodontitis: 83.3 ± 5.1  Control: 78.9 ± 4.5  7) ROS platelet levels:  Periodontitis: 0.0008 ± 4.76e-005mg of formazan/μg protein  Control: 0.001 ± 0.0002mg of formazan/μg protein  No significant difference  8) SOD activity did not differ between patients with periodontitis and control  9) Platelet catalase activity:  Periodontitis: 0.16 [0.10–0.68] U of catalase/mg protein Control: 0.16 [0.02–0.22] U of catalase/mg protein | Alterations in the intraplatelet L-arginine-NO-cGMP pathway and oxidant–antioxidant balance associated with a systemic inflammatory response may lead to platelet dysfunction. |
| *XIX. Temelli et al. (49)* | 1) MPV:  Periodontitis CAD (-): 8.2 (7.2-9.3) fL  Periodontitis CAD (+): 9.15 (6.7-11.6) fL  Control CAD (-): 7.85 (7.2-9.4) fL  Control CAD (+): 8.25 (7.1-9.6) fL  No significant difference  2) PC:  Periodontitis CAD (-)^a^: 234 (152-338) x10^9^/L  Periodontitis CAD (+)^a^: 205.5 (134-530) x10^9^/L  Control CAD (-)^a^: 234 (179-390) x10^9^/L  Control CAD (+)^a^: 221 (151-425) x10^9^/L  No significant difference | The results highlight MPV in CAD patients with or without periodontitis in terms of the relationship between inflammatory diseases and their significant low and moderate correlations with PISA values. |
| *XX. Ustaoglu et al. (55)* | 1) MPV:  Periodontitis: 8.75 ± 1.32 fL  Control: 8.22 ± 1.08 fL  p = 0.021  2) PCT:  Periodontitis: 0.223 ± 0.04 %  Control: 0.196 ± 0.04 %  p = 0.001  3) PC:  Periodontitis^a^: 258.52 ± 51.110 x10^9^/L  Control^a^: 240.48 ± 54.58 x10^9^/L  p = 0.073 | Periodontitis may elevate WBC, MPV and PCT levels compared to non-periodontitis individuals. |
| *XXI. Wang et al. (52)* | 1) PC:  Periodontitis: 252.20 ± 52.09 x10^9^/L  Control: 232.04 ± 50.95 x10^9^/L  p = 0.098  2) MPV:  Periodontitis: 9.73 ± 1.06 fL  Control: 10.24 ± 1.07 fL  p = 0.026  3) Platelet large cell ratio:  Periodontitis: 0.23 ± 0.08 %  Control: 0.28 ± 0.08 %  p = 0.007 | The decrease of MPV was related to severe periodontal inflammation and might reflect the disease activity of periodontitis. |
| *XXII. Wang et al. (46); Zhan et al. (23,24)* | 1) PC:  GAgP^b^: 230.17 ± 53.71 x10^9^/L  Control^b^: 226.89 ± 48.78 x10^9^/L  P^c^ = 0.55  2) MPV:  GAgP^b^: 9.14 ± 1.03 fL  Control^b^: 9.39 ± 1.05 fL  P^c^ = 0.03  3) PDW:  GAgP^b^: 11.22 ± 1.71 fL  Control^b^: 11.87 ± 1.72 fL  p^c^ = < 0.001  4) Platelet large cell ratio:  GAgP^b^: 0.20 ± 0.07  Control^b^: 0.23 ± 0.08  p^c^ = < 0.001 | Platelet size was reduced in GAgP patients compared to healthy controls, possibly due to the consumption of large platelets at sites of periodontal inflammation.  The G allele of the CYP1A1 rs1048943 gene was associated with GAgP, periodontal status and platelet- related inflammation status in a Chinese population.  Platelets may be involved in host responses to periodontal infection in GAgP. |
| *XXIII. Zhao et al. (54)* | PC:  Periodontitis: 228.00 (195.00, 269.00) x10^9^/L  Control: 230.00 (197.00, 271.00) x10^9^/L  p = 0.4829 | The study revealed no statistically noteworthy disparity in platelet levels between patients with periodontitis and individuals without. |

*A. actinomycetemcomitans*: *Aggregatibacter actinomycetemcomitans*, AgP: Aggressive Periodontitis, CAD: Coronary Artery Disease, CAL: Clinical Attachment Levels , CD40: Cluster of Differentiation 40, CD40L: Cluster of Differentiation 40 Ligand, cGMP: cyclic Guanosine Monophosphate, CP: Chronic Periodontitis, CVDs: Cardio Vascular Diseases, CYP1A1: cytochrome P450, family 1, subfamily A, polypeptide 1, DM: Diabetes Mellitus, eNOS: endothelial Nitric Oxide Synthase, GAgP: Generalized Aggressive Periodontitis, GPIIb/IIIa: Glycoprotein IIb/IIIa (also known as integrin αII_b_β3), GTP: Guanosine Triphosphate, iNOS: inducible Nitric Oxide Synthase, MFI: Mean Fluorescence Intensity, MPV: Mean Platelet Volume, NOS: Nitric Oxide Synthase, PAC-1: first Procaspase Activating Compound, PC: Platelet Count, PCT: Plateletcrit, PDW: Platelet Distribution Width, PF4: Platelet Factor 4, *P.* gingivalis: *Porphyromonas* gingivalis, PISA: Periodontal Inflammatory Surface Area, PLCR: Platelet Large Cell Ratio, Rho: Ras homolog, ROS: Reactive Oxygen Species, sCD40L: soluble Cluster of Differentiation 40 Ligand, SOD: Superoxide Dismutase, sP-selectin: soluble P-selectin, *T.* forsythia: *Tannerella* forsythia, VASP: Vasodilator-Stimulated Phosphoprotein, WBC: White Blood Cell.

a: converted to x10^9^/L from the authors of the present review, b: provided by the original authors, c: calculated by the authors of the present review (Welch t test).

**Appendix 8**

Meta-analysis of selected studies for platelet count


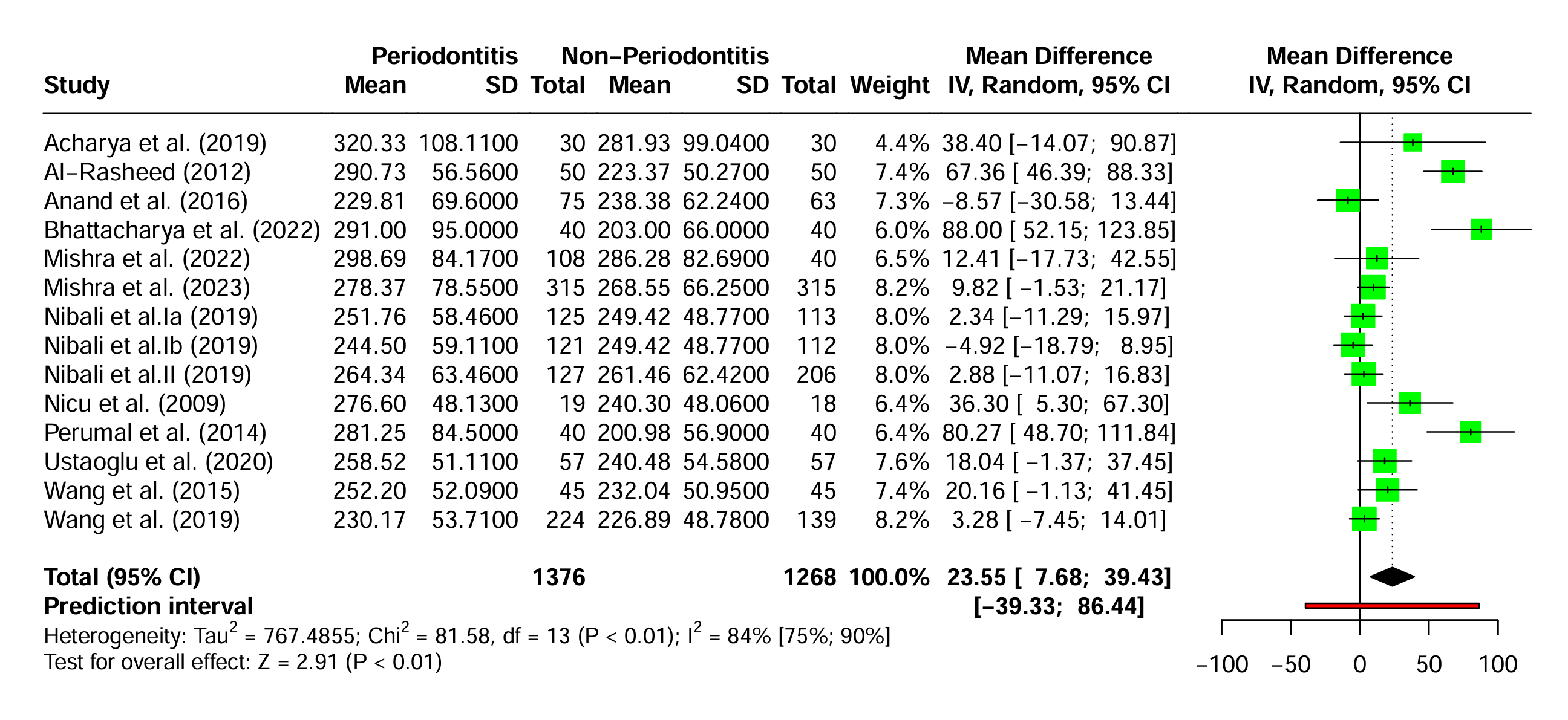


**Appendix 9**

Sub-analyses for platelet count of selected studies based on periodontitis case definition

A. Studies including patients with molar/incisor or generalized stage 3-4, grade C due to rapid progression and/or early onset periodontitis


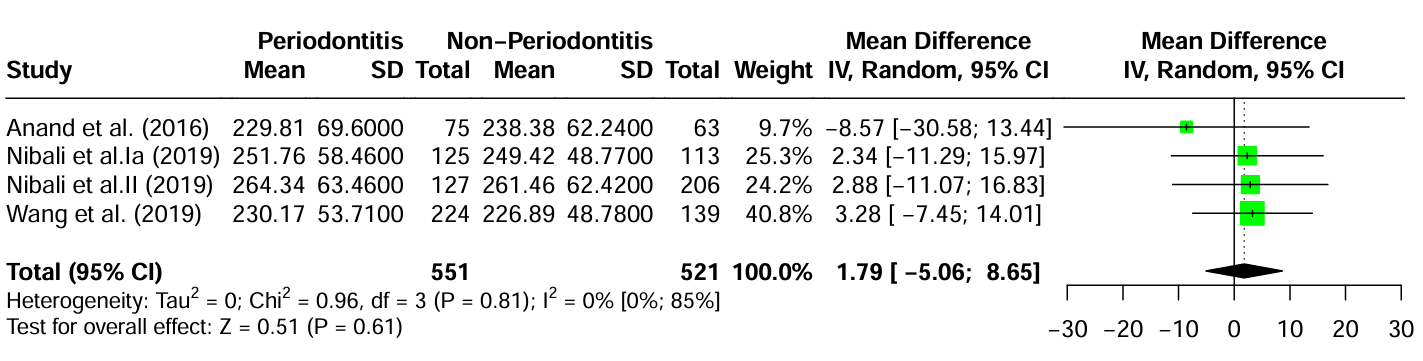


B. Studies including patients with non-molar/incisor or generalized stage 3-4, grade C due to rapid progression and/or early onset periodontitis


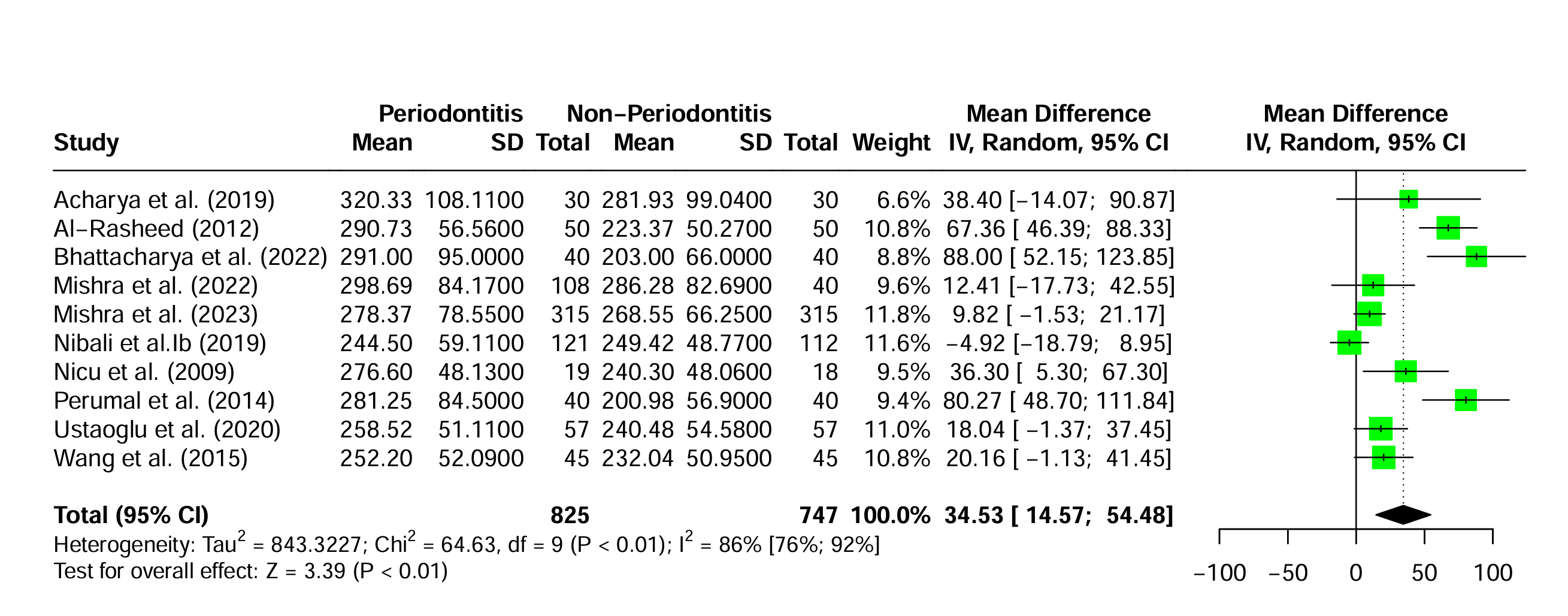


**Appendix 10**

Sub-analyses for platelet count of selected studies based on risk of bias

A. Studies with low risk of bias


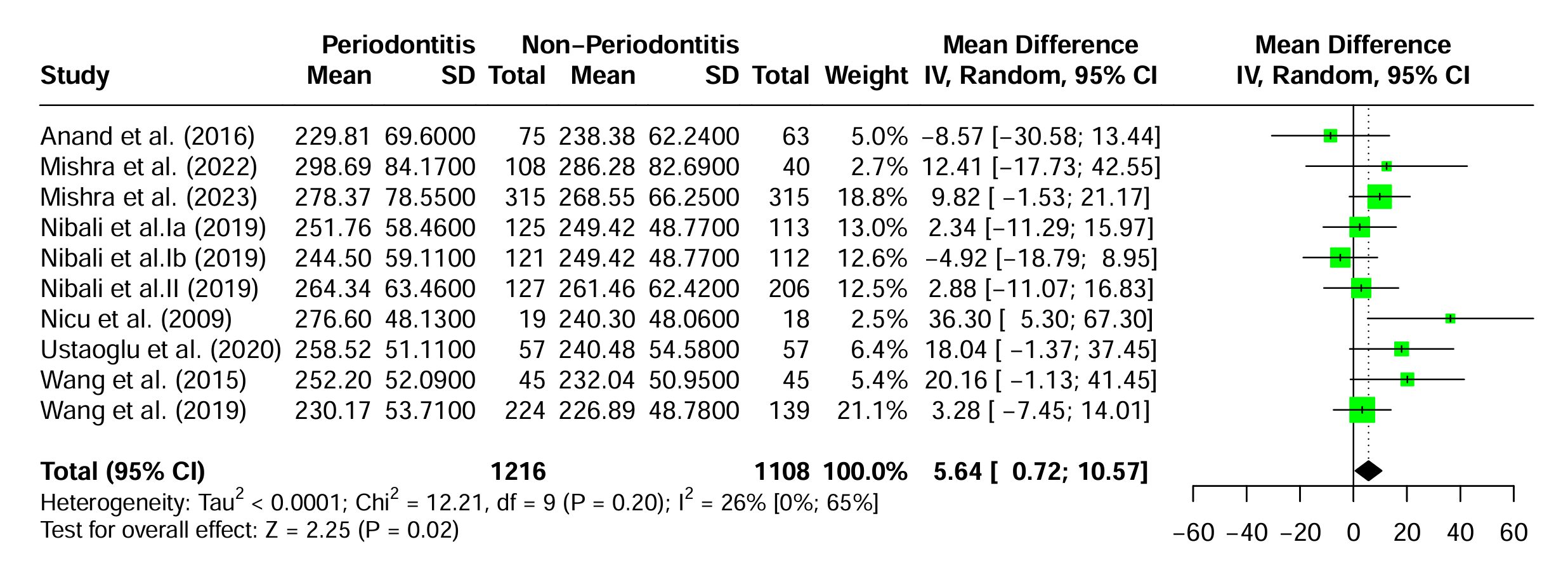


B. Studies with moderate risk of bias


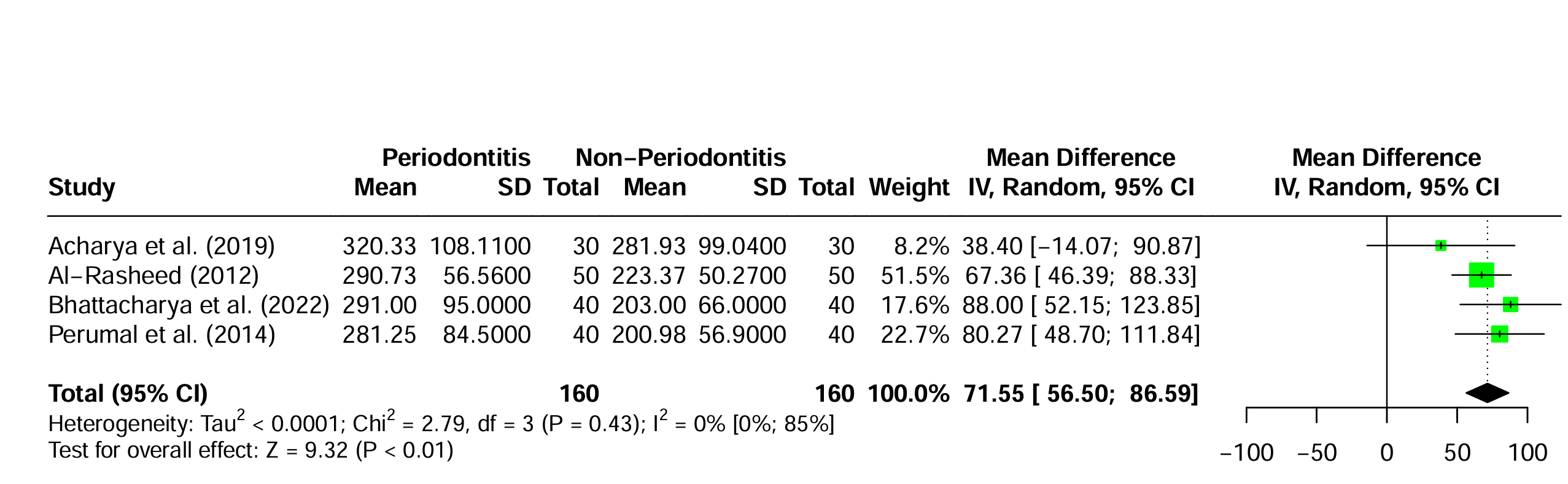


**Appendix 11**

Sub-analyses for platelet count of selected studies based on study design

A. Studies with case-control design


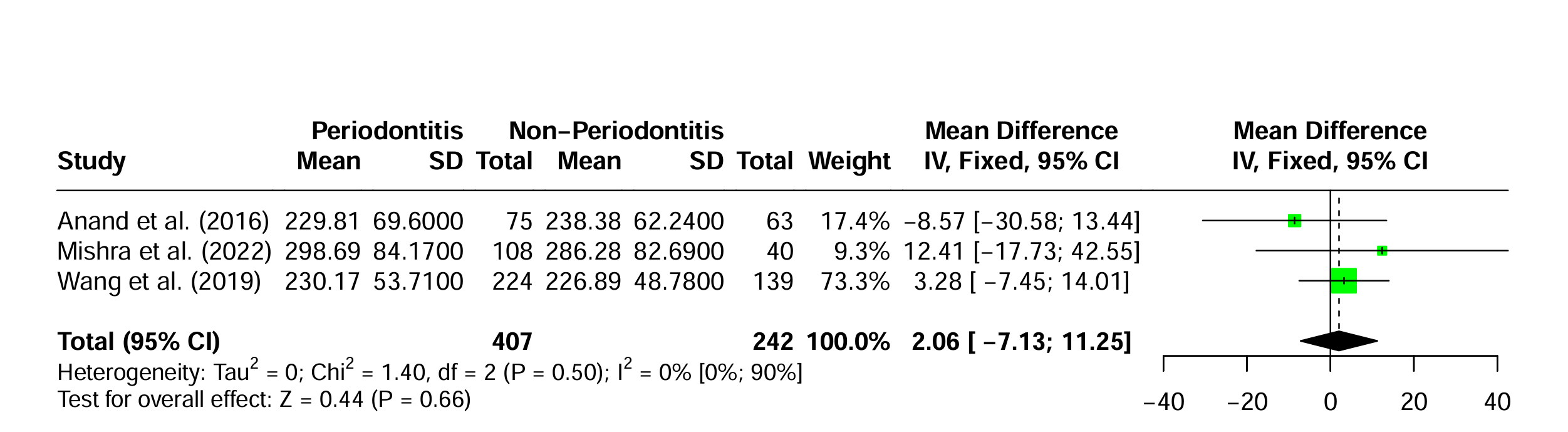


B. Studies with cross-sectional design


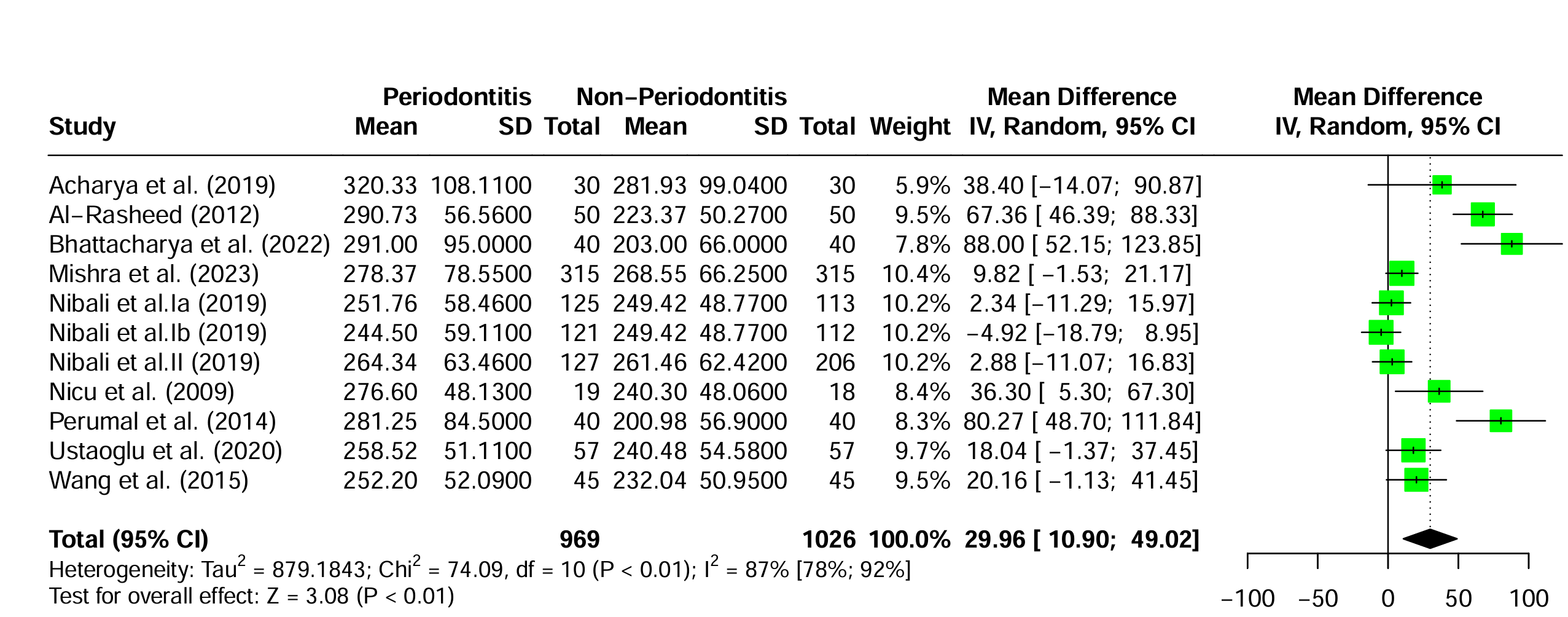


**Appendix 12**

Sub-analyses for platelet count of selected studies based on smoking status of participants

A. Studies including smokers


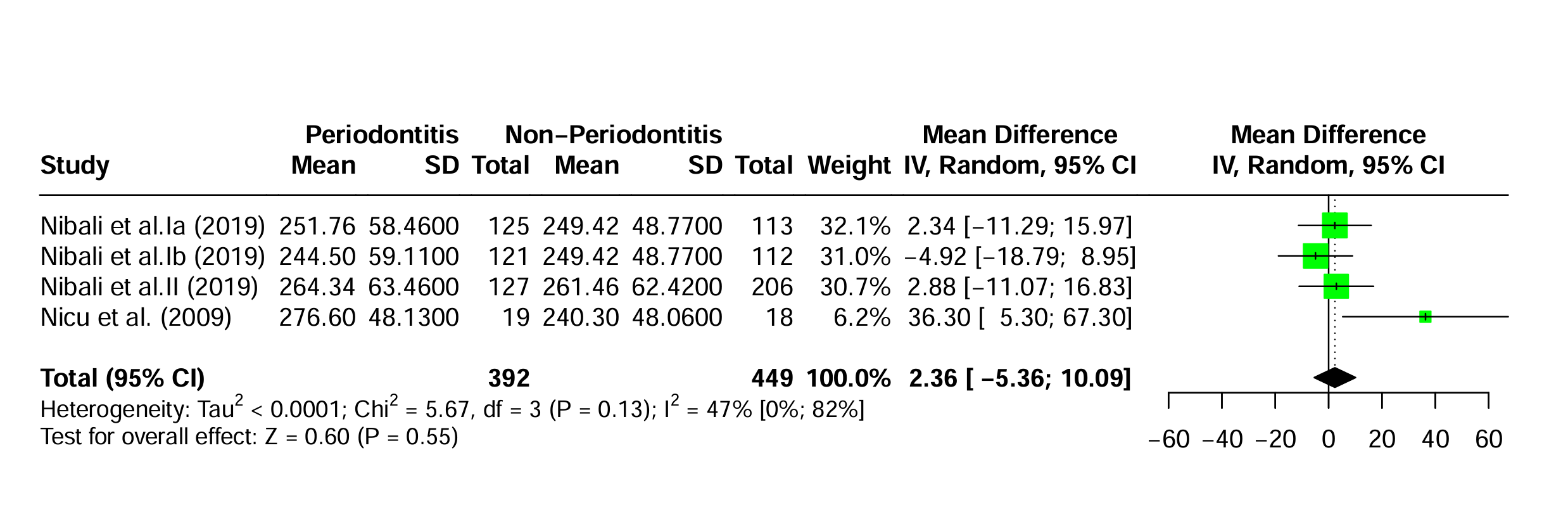


B. Studies excluding smokers


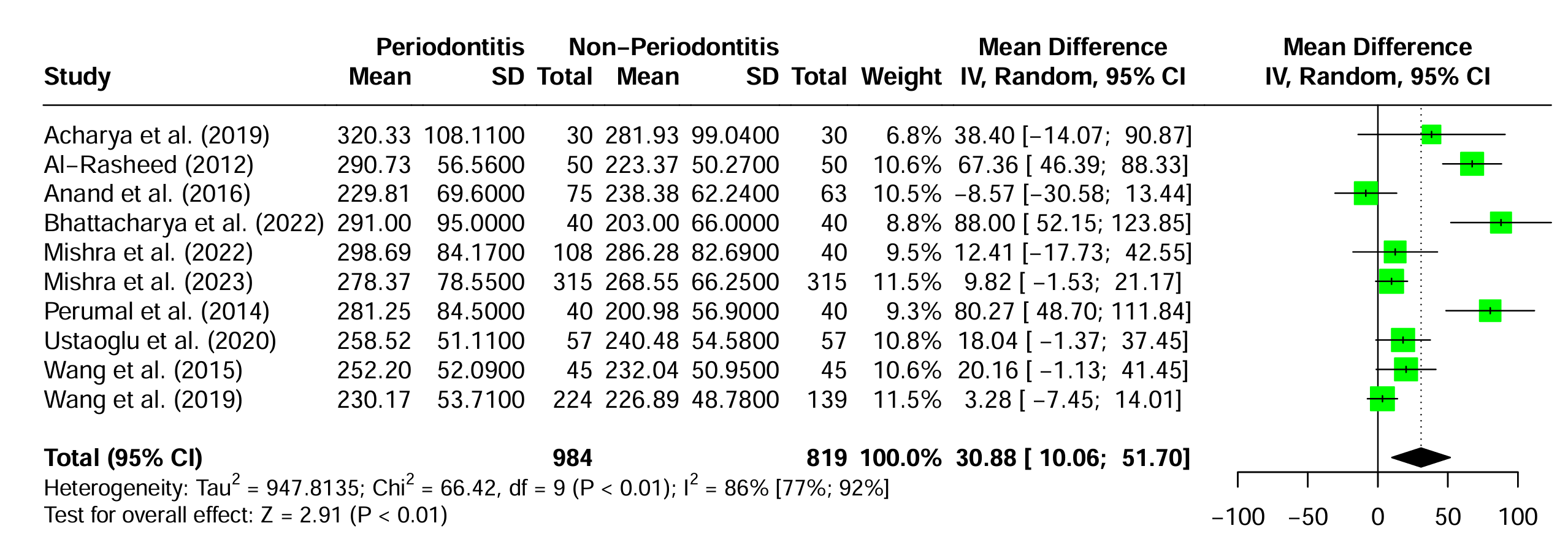


**Appendix 13**

Funnel plot of selected studies for platelet count

**
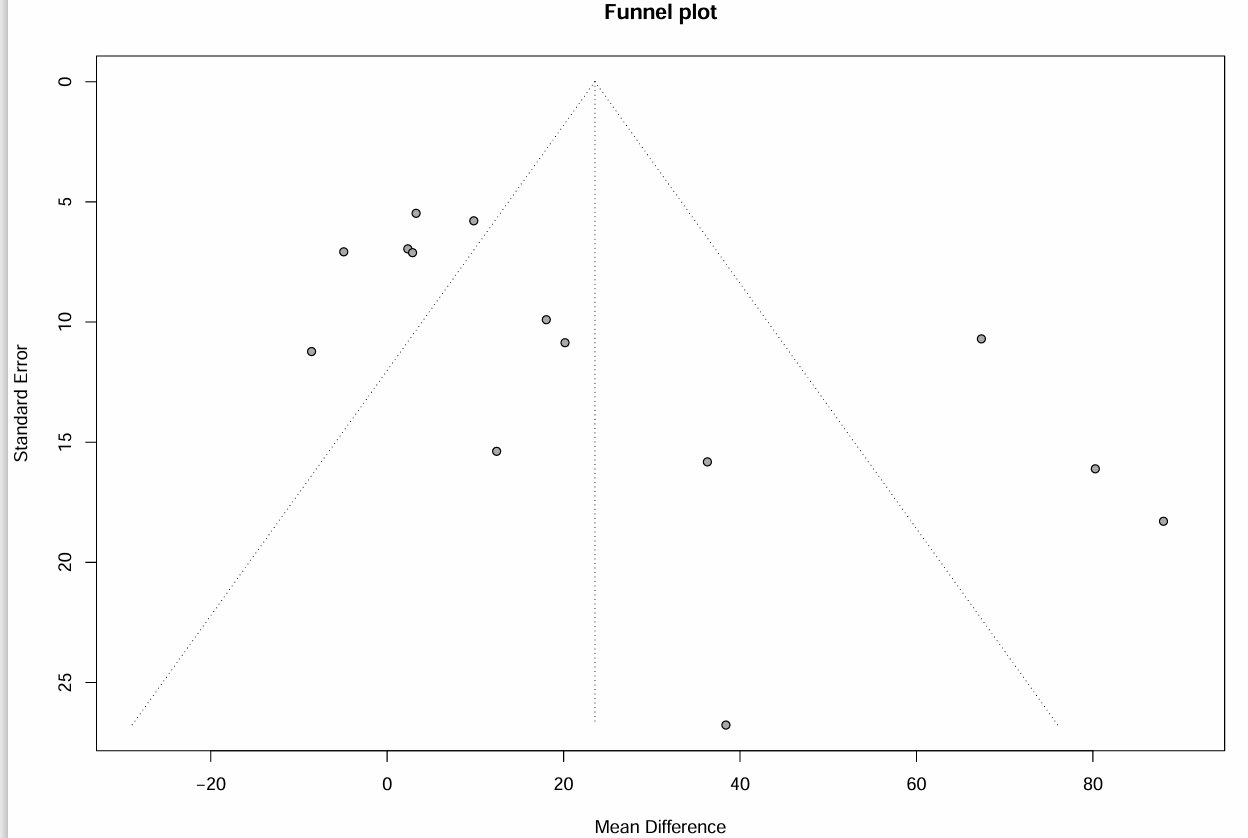
**

**Appendix 14**

Meta-regression on participants age through selected studies for platelet count, p = 0.28


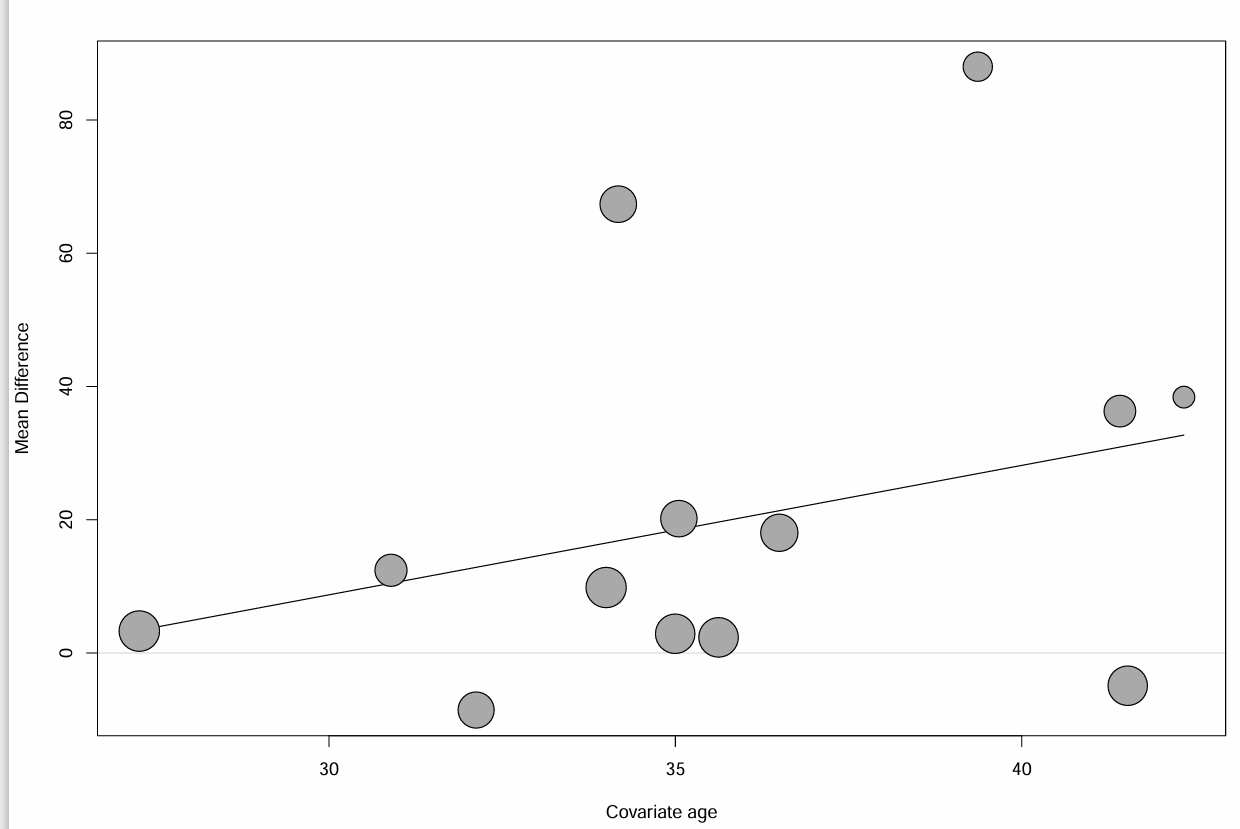


**Appendix 15**

Meta-regression on participants females ratio through selected studies for platelet count, p = 0.18


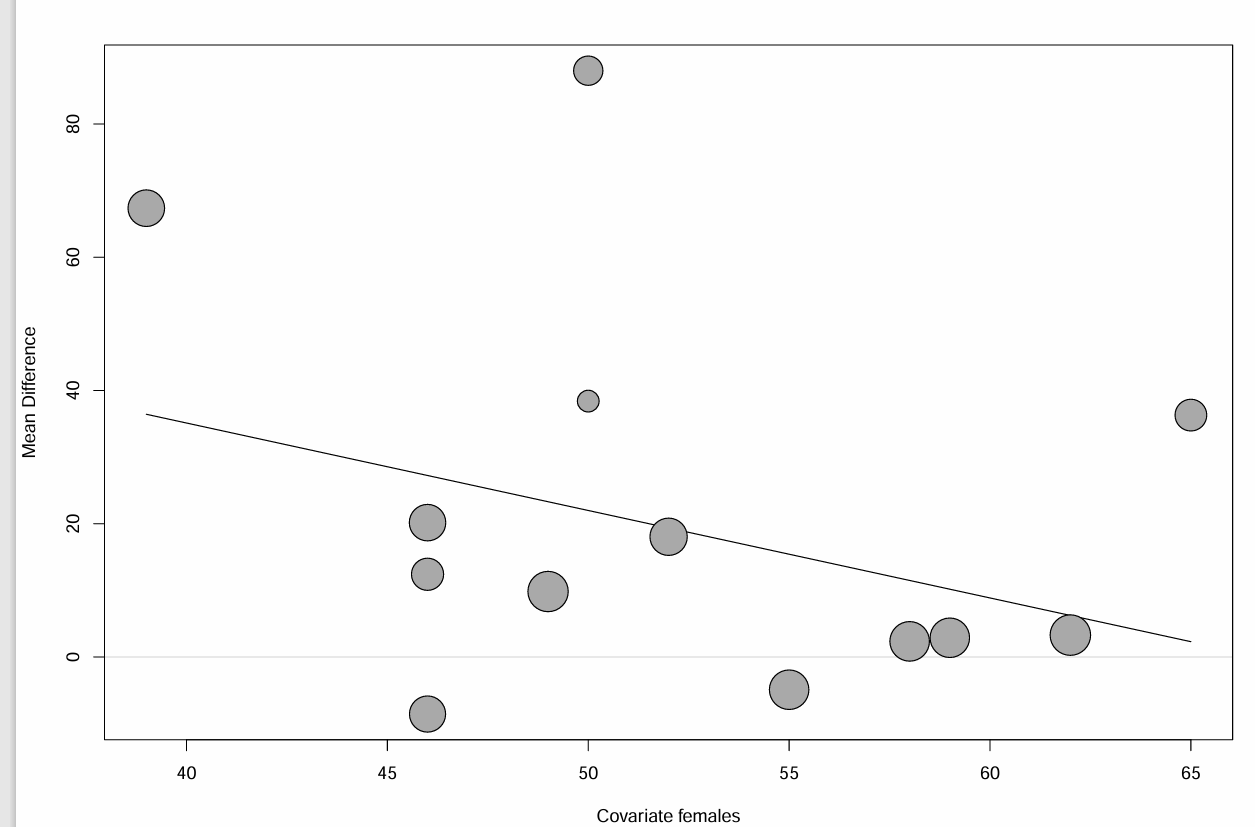


**Appendix 16**

Meta-analysis of selected studies for mean platelet volume


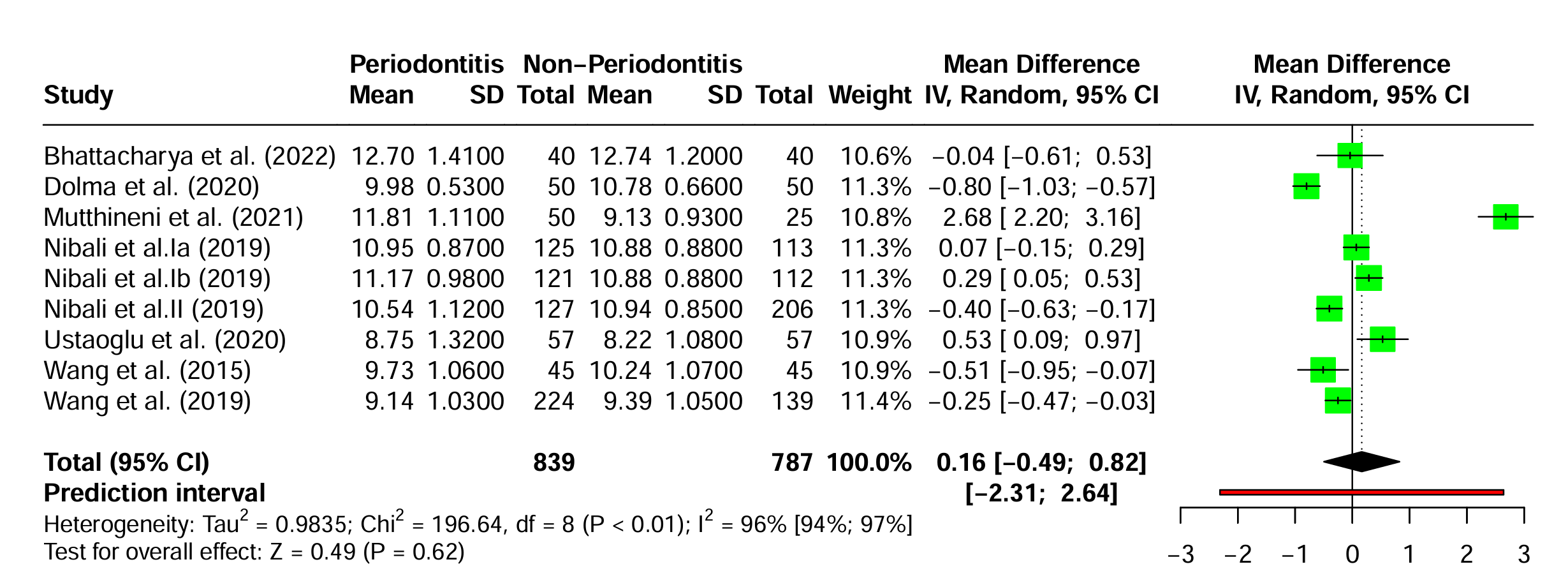


**Appendix 17**

Sub-analyses for mean platelet volume of selected studies based on periodontitis case definition

A. Studies including patients with molar/incisor or generalized stage 3-4, grade C due to rapid progression and/or early onset periodontitis


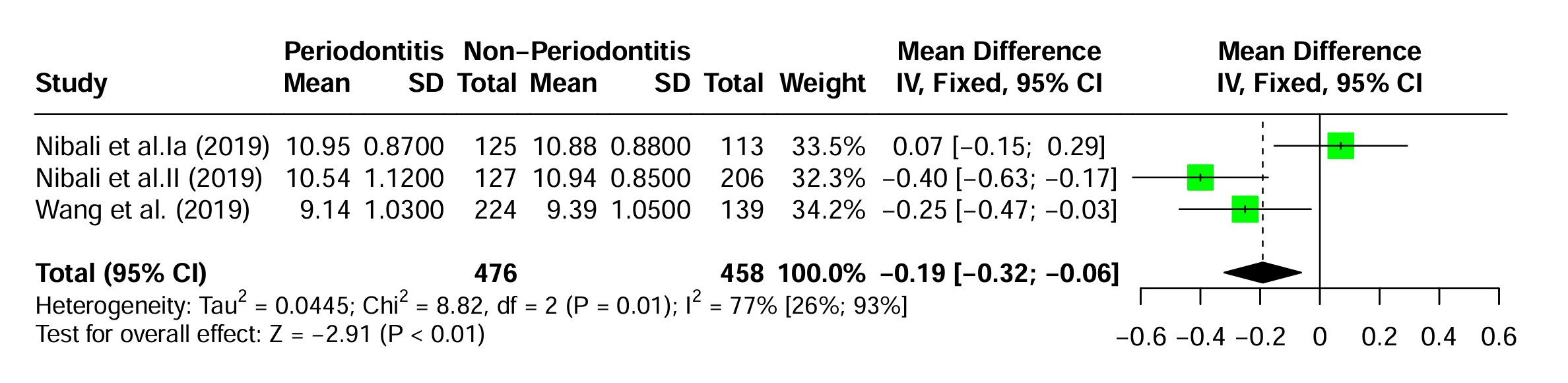


B. Studies including patients with non-molar/incisor or generalized stage 3-4, grade C due to rapid progression and/or early onset periodontitis


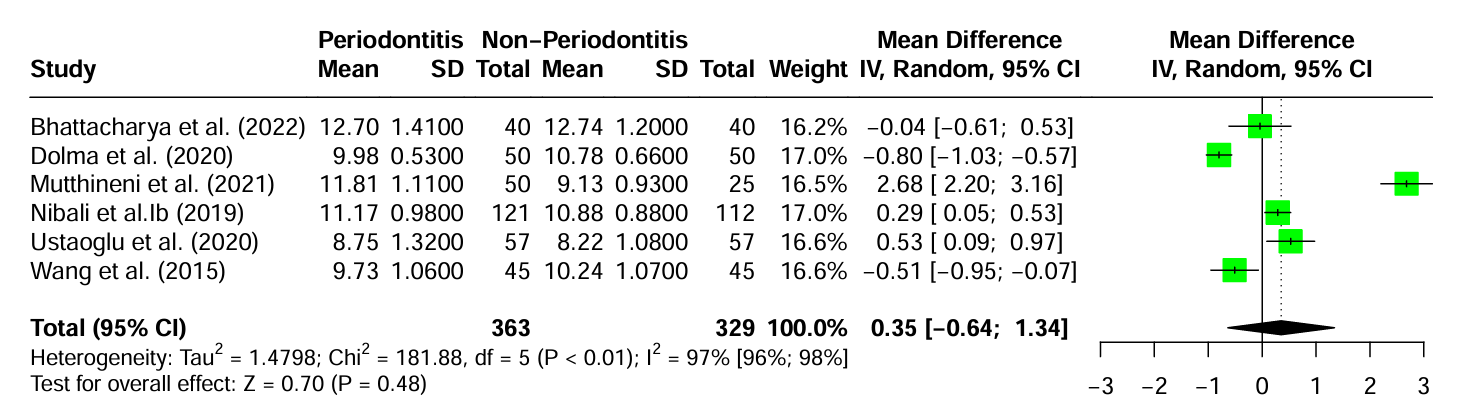


**Appendix 18**

Sub-analyses for mean platelet volume of selected studies based on risk of bias

A. Studies with low risk of bias


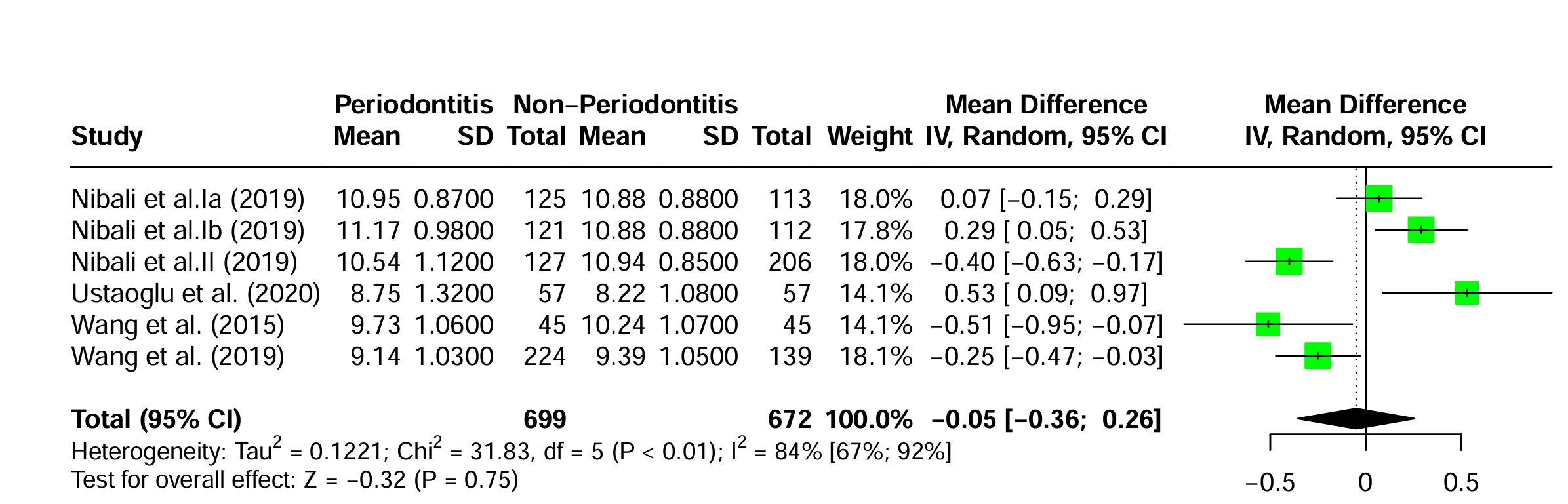


B. Studies with moderate risk of bias


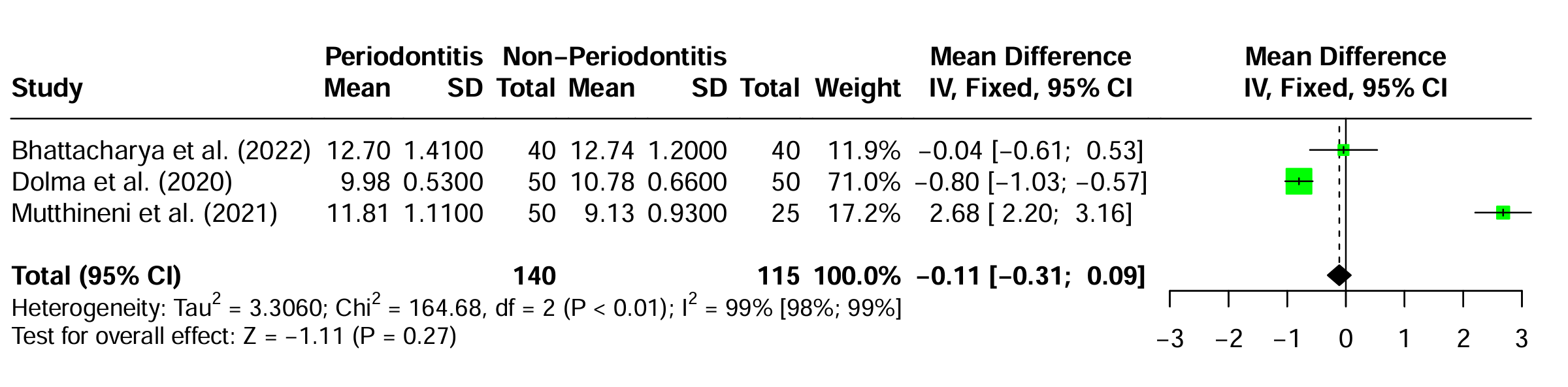


**Appendix 19**

Sub-analyses for mean platelet volume of selected studies based on study design

Studies with cross-sectional design


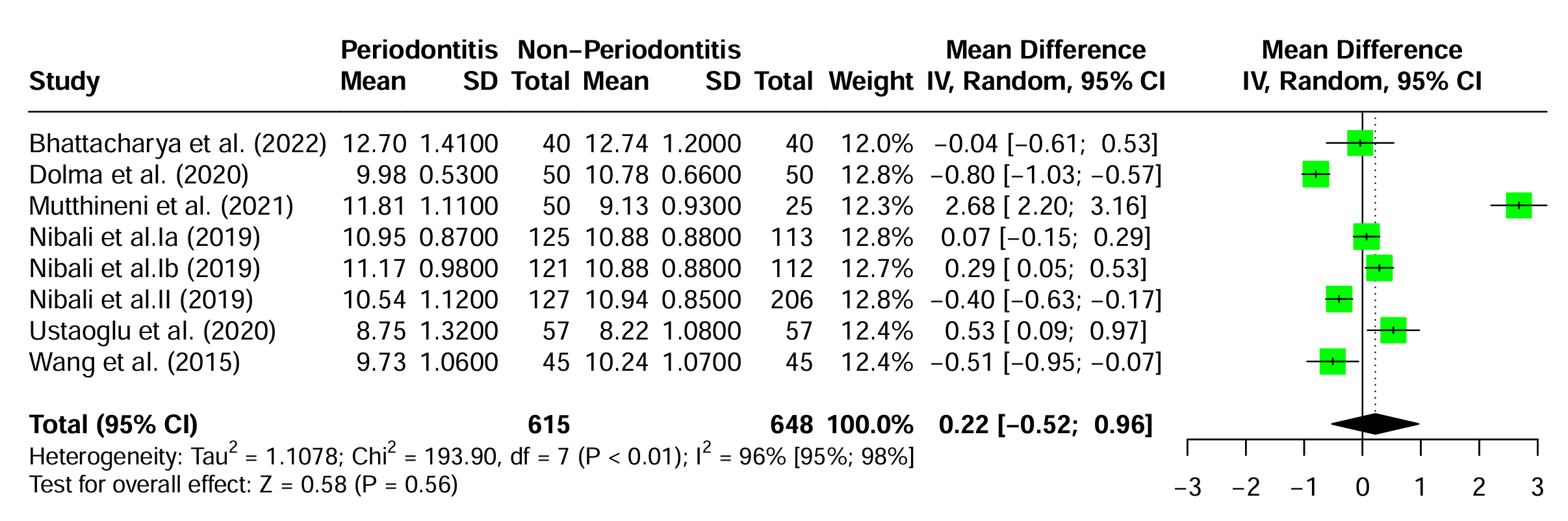


**Appendix 20**

Sub-analyses for mean platelet volume of selected studies based on smoking status of participants

A. Studies including smokers


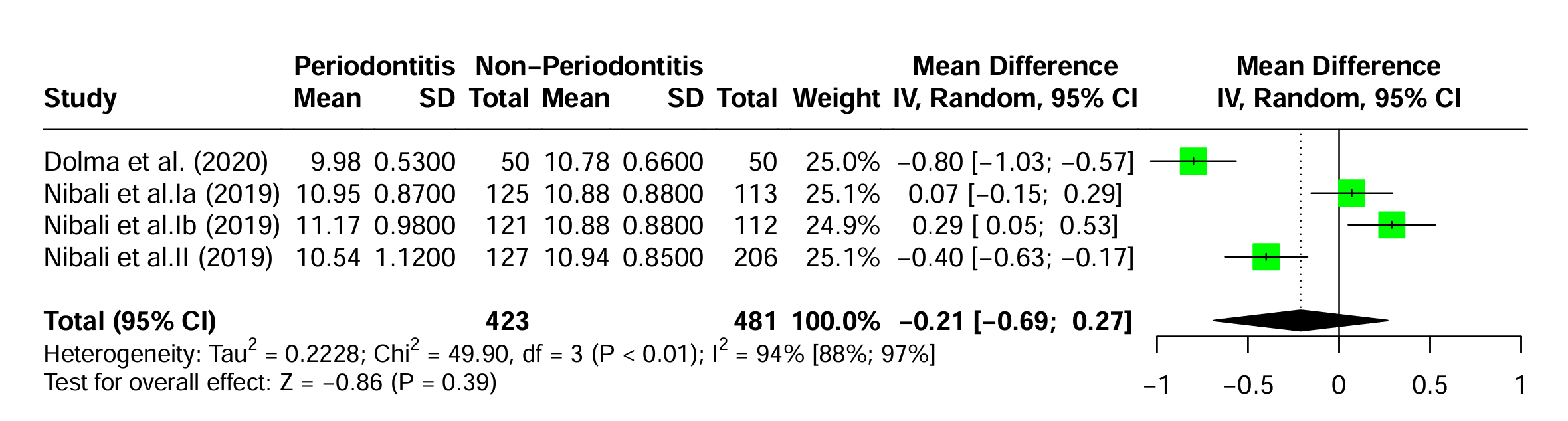


B. Studies excluding smokers


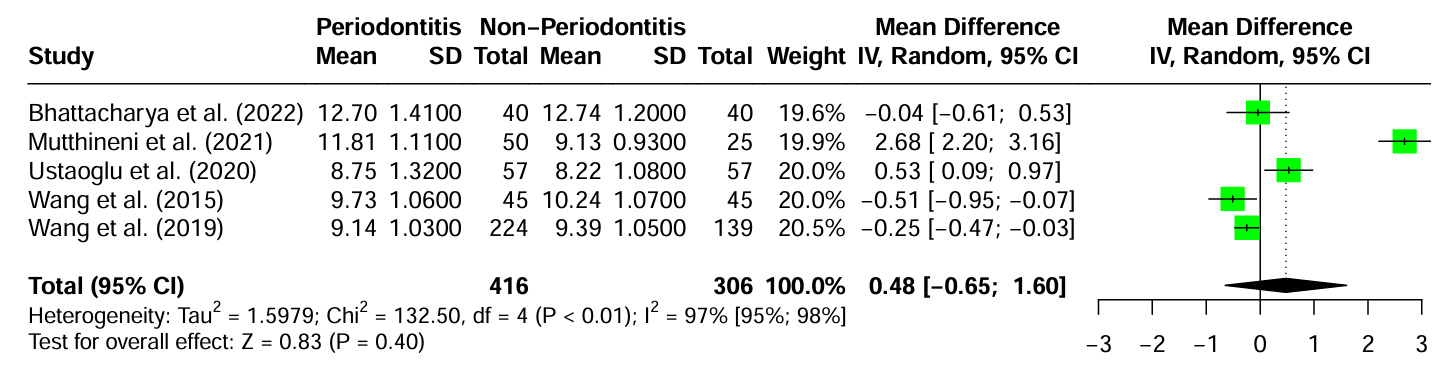


**Appendix 21**

Doi plot of selected studies for mean platelet volume


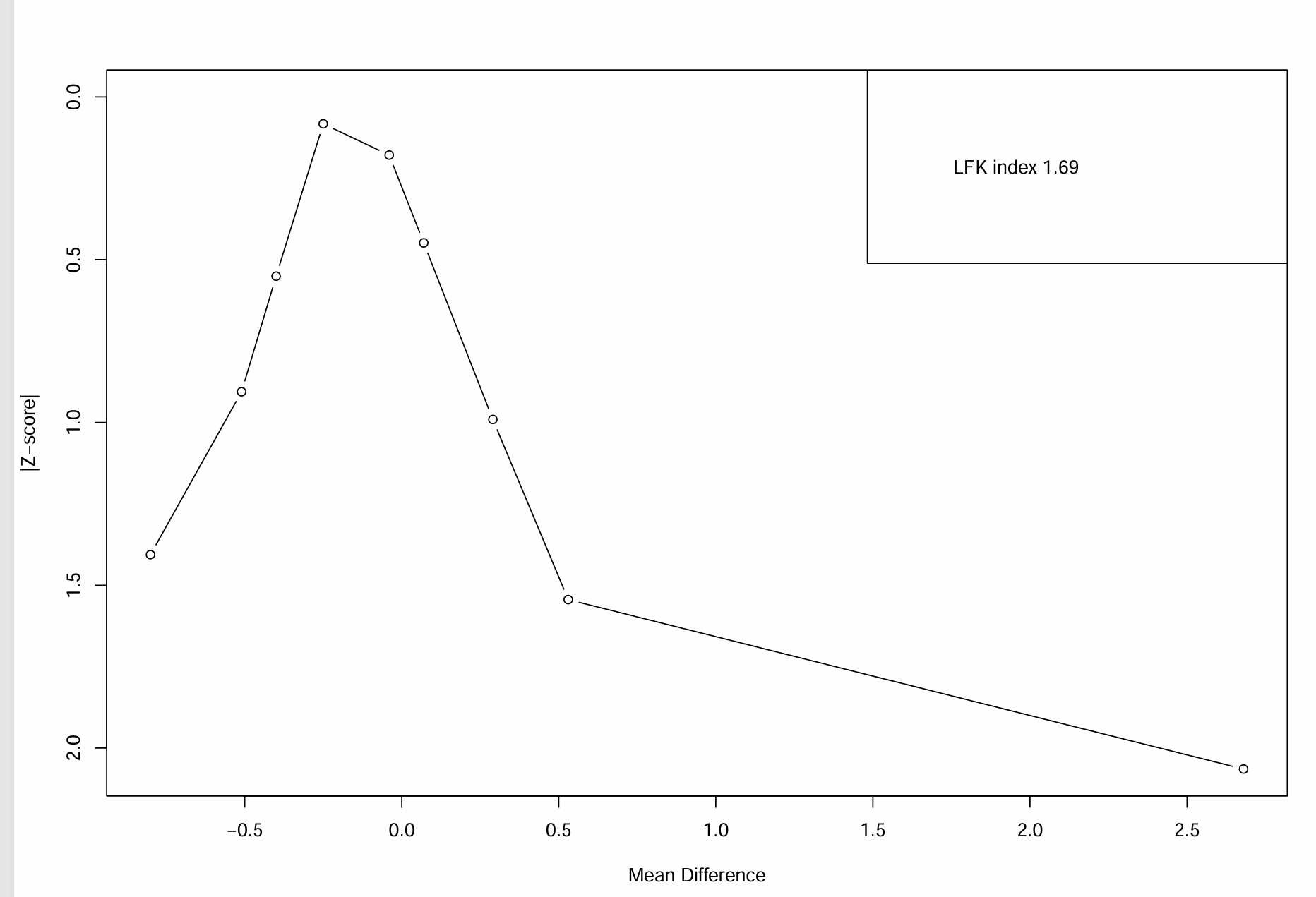

Supplement: Supplementary file 1 — Appendix S1.–S21. [file JRE-60-872-s001.docx]
